# Advancing genetic improvement in the omics era: status and priorities for United States aquaculture

**DOI:** 10.1186/s12864-025-11247-z

**Published:** 2025-02-17

**Authors:** Linnea K. Andersen, Neil F. Thompson, Jason W. Abernathy, Ridwan O. Ahmed, Ali Ali, Rafet Al-Tobasei, Benjamin H. Beck, Bernarda Calla, Thomas A. Delomas, Rex A. Dunham, Christine G. Elsik, S. Adam Fuller, Julio C. García, Mackenzie R. Gavery, Christopher M. Hollenbeck, Kevin M. Johnson, Emily Kunselman, Erin L. Legacki, Sixin Liu, Zhanjiang Liu, Brittany Martin, Joseph L. Matt, Samuel A. May, Caitlin E. Older, Ken Overturf, Yniv Palti, Eric J. Peatman, Brian C. Peterson, Michael P. Phelps, Louis V. Plough, Mark P. Polinski, Dina A. Proestou, Catherine M. Purcell, Sylvie M. A. Quiniou, Guglielmo Raymo, Caird E. Rexroad, Kenneth L. Riley, Steven B. Roberts, Luke A. Roy, Mohamed Salem, Kelly Simpson, Geoffrey C. Waldbieser, Hanping Wang, Charles D. Waters, Benjamin J. Reading, on behalf of The Aquaculture Genomics, Genetics and Breeding Workshop

**Affiliations:** 1 USDA-ARS Aquatic Animal Health Research Unit, Auburn, AL USA; 2 USDA-ARS Pacific Shellfish Research Unit, Newport, OR USA; 3 Department of Animal and Avian Sciences, University of Maryland, College Park, MD USA; 4 Middle Tennessee State University, Murfreesboro, TN USA; 5 USDA-ARS National Cold Water Marine Aquaculture Center, Kingston, RI USA; 6 School of Fisheries, Aquaculture, and Aquatic Sciences, Auburn University, Auburn, AL USA; 7 University of Missouri, Columbia, MO USA; 8 USDA-ARS Harry K Dupree Stuttgart National Aquaculture Research Center, Stuttgart, AR USA; 9 Environmental and Fishery Sciences Division, NOAA Northwest Fisheries Science Center, Seattle, WA USA; 10 Texas A&M AgriLife Research, College Station, TX USA; 11 Texas A&M University - Corpus Christi, Corpus Christi, TX USA; 12 California Sea Grant, Scripps Institution of Oceanography, University of California San Diego, La Jolla, CA USA; 13 Biological Sciences Department, Center for Coastal Marine Sciences, California Polytechnic State University, San Luis Obispo, CA USA; 14 Hubbs-SeaWorld Research Institute, San Diego, CA USA; 15 USDA-ARS National Cold Water Marine Aquaculture Center, Orono, ME USA; 16 USDA-ARS National Center for Cool and Cold Water Aquaculture, Kearneysville, WV USA; 17 Department of Biology, Tennessee Technological University, Cookeville, TN USA; 18 USDA-ARS Warmwater Aquaculture Research Unit, Stoneville, MS USA; 19 USDA-ARS Small Grains and Potato Germplasm Research, Hagerman, ID USA; 20 Harvest Select Catfish Inc, Inverness, MS USA; 21 Washington State University, Pullman, WA USA; 22 Horn Point Laboratory, University of Maryland Center for Environmental Science, Cambridge, MD USA; 23 NOAA Fisheries, Southwest Fisheries Science Center, La Jolla, CA USA; 24 USDA-ARS Office of National Programs, Beltsville, MD USA; 25 Office of Aquaculture, NOAA Fisheries, Silver Spring, MD USA; 26 University of Washington, Seattle, WA USA; 27 School of Fisheries, Aquaculture, and Aquatic Sciences, Auburn University, Alabama Fish Farming Center, Greensboro, AL USA; 28 The Ohio State University, Columbus, OH USA; 29 NOAA Alaska Fisheries Science Center Auke Bay Laboratories, Juneau, AK USA; 30 Department of Applied Ecology, North Carolina State University, Raleigh, NC USA

**Keywords:** Aquaculture, Genomics, Genetics, Breeding, Genome-to-phenome, Multi-omics data, Phenomics, Interdisciplinary integration, Gene-editing, Education and workforce training

## Abstract

**Background:**

The innovations of the “Omics Era” have ushered in significant advancements in genetic improvement of agriculturally important animal species through transforming genetics, genomics and breeding strategies. These advancements were often coordinated, in part, by support provided over 30 years through the 1993–2023 National Research Support Project 8 (NRSP8, National Animal Genome Research Program, NAGRP) and affiliate projects focused on enabling genomic discoveries in livestock, poultry, and aquaculture species. These significant and parallel advances demand strategic planning of future research priorities. This paper, as an output from the May 2023 Aquaculture Genomics, Genetics, and Breeding Workshop, provides an updated status of genomic resources for United States aquaculture species, highlighting major achievements and emerging priorities.

**Main text:**

Finfish and shellfish genome and omics resources enhance our understanding of genetic architecture and heritability of performance and production traits. The 2023 Workshop identified present aims for aquaculture genomics/omics research to build on this progress: (1) advancing reference genome assembly quality; (2) integrating multi-omics data to enhance analysis of production and performance traits; (3) developing resources for the collection and integration of phenomics data; (4) creating pathways for applying and integrating genomics information across animal industries; and (5) providing training, extension, and outreach to support the application of genome to phenome. Research focuses should emphasize phenomics data collection, artificial intelligence, identifying causative relationships between genotypes and phenotypes, establishing pathways to apply genomic information and tools across aquaculture industries, and an expansion of training programs for the next-generation workforce to facilitate integration of genomic sciences into aquaculture operations to enhance productivity, competitiveness, and sustainability.

**Conclusion:**

This collective vision of applying genomics to aquaculture breeding with focus on the highlighted priorities is intended to facilitate the continued advancement of the United States aquaculture genomics, genetics and breeding research community and industries. Critical challenges ahead include the practical application of genomic tools and analytical frameworks beyond academic and research communities that require collaborative partnerships between academia, government, and industry. The scope of this review encompasses the use of omics tools and applications in the study of aquatic animals cultivated for human consumption in aquaculture settings throughout their life-cycle.

**Supplementary Information:**

The online version contains supplementary material available at 10.1186/s12864-025-11247-z.

## Background

The “Omics Era” has facilitated the development of genome-enabled breeding strategies by allowing scientists to routinely generate individual sequence data and assess the relationships between genetic, genomic, and phenotypic variations across production systems of agriculturally important species. Aquaculture is agriculture and one food production sector that has and continues to benefit greatly from the omics revolution in multiple ways, ranging from direct impacts on the propagated organisms, and indirectly through understanding the pests, pathogens, and aquatic environment that influence production efficiencies [1]. Recent advancements in aquaculture genomics have been driven by technological and methodological innovations. In the United States (U.S.) these research contributions have largely been facilitated via the National Research Support Project 8 (NRSP8) National Animal Genome Research Program (NAGRP)^1^ for U.S. agricultural animal species (1993–2023) and complementary research projects supported by United States Department of Agriculture (USDA) Agricultural Research Service (ARS) and USDA National Institute of Food and Agriculture (NIFA), among other organizations. The aquaculture component of the NRSP8 program began in 2003 and focused on species that had established industries (i.e., existing producers and markets) during the funding period. The NRSP8 provided a forum for aquaculture scientists to network, form collaborations, facilitate and implement large-scale projects, offer feedback to funding agencies on national research priorities, provide students with an opportunity to present research, engage with international crop and animal scientists, and seek future employment opportunities in aquaculture genomics and related areas of study (i.e., omics).

**Fig. 1:**
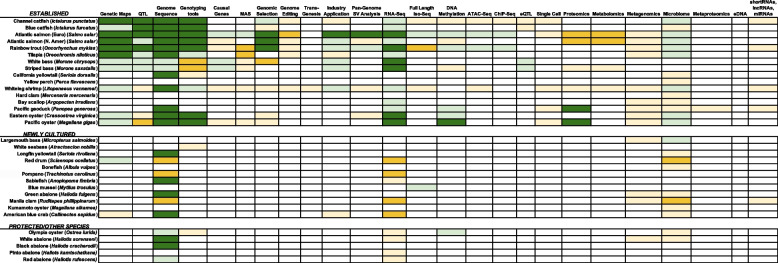
Current status of omics resources for species cultivated in United States aquaculture. The current status is indicated with various colors: Dark green: good status; light green, outstanding progress has been made, but additional work still needed; dark yellow: significant progress has been made, but significant amount of additional work still needed; light yellow, some progress has been made

Since the last comprehensive assessment of the U.S. aquaculture genomics, genetics and breeding sector [2], there has been considerable advancement within a ‘Science to Practice’ framework [3]. Specifically, a focus was placed on developing “infrastructure,” including genomics and bioinformatics tools, databases, and genetic resources, to enable genomics-oriented discovery science that informs on-the-ground breeding and production of aquaculture animals. Figure 1 presents the current status of omics resources that have been generated for U.S. aquaculture species, with a focus on species with established industries (“Established”) in addition to species with emergent industries (“Newly Cultured”) and those cultured as a means of protecting wild populations (“Protected”). The aquaculture genomics community achieved this infrastructure goal across a broad range of vertebrate and invertebrate species propagated in freshwater and marine environments through the generation of new or vastly improved reference genome assemblies, genotyping arrays, and, for some species in particular, an expansive suite of high-throughput omics tools (Fig. 1; citations associated with each species and category are provided in File S1).

Interrogation and use of whole genome data as an anchor for a multitude of applications (e.g., genomic selection, GS; marker-assisted selection, MAS; genetic engineering, population structure analysis, demographic monitoring) has occurred in several finfish and shellfish species [4–9]. Finfish species with large economies of scale (e.g., salmonids) have experienced rapid advancements that have directly impacted commercial industry due to improved genetic infrastructure. These advancements include applying genome and omics data to improve growth [10–12], develop disease resistant lines [13–16], achieve monosex and sterilization [17, 18], evaluate environmental pollution burdens [19], and enhance seafood traceability [20, 21]*.* Impactful advancements have also been made within smaller scale and newly cultured species, such that improved germplasm created using novel, and continually developing, omics-based tools are available to commercial industries as they expand [22]. Beyond the aquaculture species, omics applications to studying pests and pathogens have enabled vaccine development [23], and studies of microbiota that inhabit the host and the aquatic environment have led to improvements in animal production and production system operation [24–27]. In all, the rapid genome infrastructure advancements made to date have enabled the exploration of the genetic architecture associated with production traits, allowing for the more precise evaluation of key quantitative genetic parameters and assessment of artificial selection potential.

While it is clear the aquaculture genetics, genomics, and breeding community has made significant strides since the last comprehensive review [2], new priorities and needs have emerged. Genetic resource development remains a continuous need within aquaculture due to the high diversity of cultured species [28]. There has been a growing focus on data integration strategies that facilitate trait dissection (e.g., genotype-to-phenotype, G2P, associations) within programs and among species, although this has been especially challenging as the number and dimensionality of data have grown exponentially with the rapid and continuous development of advanced technologies. The aquaculture genomics community has been highly successful in continuing to expand libraries of knowledge (data and analyses), but integration of these data and adoption of new tools (e.g., artificial intelligence, AI, and machine learning, ML) are hurdles that require technological cost reduction, improved accessibility of data and methodologies, and teaching and training of practitioners at multiple levels. Indeed, convergence of AI/ML and genomics will be a major component of the future of agriculture, including aquaculture [29]. Moreover, the practical application of these data and analytical frameworks to industry programs outside of the academic research community is contingent on teaching and training the next-generation workforce. Doing so is critical as we enter a “post-genomics era” in which these tools have been developed, are readily available, and require educated and trained personnel to apply these advancements towards industry priorities.

Here we update the current state of genomic resources for established and newly cultured aquaculture species in the U.S. and outline future directions and priorities for the aquaculture omics research community. The update is organized by the present aims of the aquaculture omics community, with examples of achievements that have been made within each aim and descriptions of components that have yet to be realized. These aims are: (1) advancing reference genome assembly quality; (2) integrating multi-omics data to enhance analysis of production and performance traits; (3) developing resources for the collection and integration of phenomics data; (4) creating pathways for applying and integrating genomics information across animal industries; and (5) providing training, extension, and outreach to support the application of genome to phenome. The scope of this review is primarily on aquaculture species produced commercially in the U.S. for human consumption. Although the focus is primarily on U.S.-based aquaculture research pertaining to food production, references to research conducted for fisheries management and conservation and the international community are included in cases where this research represents a tool, technique, or method that is utilized by U.S.-based aquaculture researchers, or the work represents the leading-edge of research globally.

### Aim 1: Advancing reference genome assembly quality

Genome sequencing has progressed rapidly since the advent of next-generation sequencing, with current technology allowing for high-quality genome assemblies to be produced relatively quickly and inexpensively. A current challenge is understanding the structures and functions of genomes and how they impact phenotypes, which has guided research efforts since the publication of the 2018–2027 Genome to Phenome Blueprint [3]. The aquaculture genomics community must continue its efforts to generate high-quality genome assemblies as they facilitate research on functional variants, spatial and epigenomic regulation of performance traits, and genome-enabled breeding (i.e., GS), which have direct and indirect applications to U.S. commercial aquaculture. A high-quality genome assembly is defined by having (1) high completeness, (2) high accuracy, (3) high contiguity, (4) sequences anchored to chromosomes, (5) sex chromosome sequences (if present), and (6) haplotypes resolved. These criteria were presented as priority goals in the prior review [2] and largely encompass the ethos of telomere-to-telomere (T2T) initiatives to routinely produce phased, complete, and gapless diploid genomes. In advanced research domains, a T2T genome can be produced in hours with complete variant discovery for all chromosomes [30]. For example, there are now instances in which the initial genome assembly meets these “high-quality” criteria (e.g., Sablefish, *Anoplopoma fimbria* [31]) and we expect this to become more prevalent. However, attaining these criteria remains among the highest needs for most aquaculture species.

References for the most recent genome assemblies for established and newly cultured aquaculture species are provided in Table 1. This list includes genome assemblies accessible through National Center for Biotechnology Information (NCBI) GenBank® and indicates if presently included in AquaMine (v1.2; https://aquamine.elsiklab.missouri.edu/, Elsik Lab, University of Missouri), a data mining system that facilitates integration and comparison of genomic data across species. There are too many genome assemblies to comprehensively cover within the text, however, examples of recent advancements toward improving genome assemblies and meeting the “high-quality” criteria include: anchoring sequences to chromosomes in Pacific oyster (*Magallana gigas*) [32, 33], eastern oyster (*Crassostrea virginica*) [34], Atlantic salmon (*Salmo salar*) [35], Nile tilapia (*Oreochromis niloticus*) GIFT strain [36], and Pacific white shrimp (*Litopenaeus vannamei*) [37]; improving genome assembly contiguity and sequencing the sex determination gene on the Y chromosome of rainbow trout (*Oncorhynchus mykiss*) [38]; and reducing gaps in chromosomes and scaffold assemblies in blue (*Ictalurus furcatus*) and channel catfish (*Ictalurus punctatus*) [39–41].

**Table 1:** Current reference genome assemblies of important U.S. aquaculture species published through NCBI GenBank®. An asterisk (*) indicates the assembly is presently included in the data mining system AquaMine (v1.2; https://aquamine.elsiklab.missouri.edu/; Elsik Lab, University of Missouri) or planned for the next release (**)

Species	Common name	Assembly	Genbank	Reference
Anoplopoma fimbria**	Sablefish	Afim_UVic_2022	GCA_027596085.2	Flores et al., 2023 [31]
Clupea harengus*	Atlantic herring	Ch_v2.0.2	GCA_900700415.2	Pettersson et al., 2019 [42]
Coregonus clupeaformis*	Lake whitefish	ASM2061545v1	GCA_020615455.1	Mérot et a. 2023 [43]
Crassostrea virginica*	Eastern oyster	C_virginica-3.0	GCA_002022765.4	Gómez-Chiarri et al., 2015 [44]
Esox lucius*	Northern pike	Eluc_v4	GCA_004634155.1	Rondeau et al., 2014 [45]
Esox lucius**	Northern pike	fEsoLuc1.pri	GCA_011004845.1	Rhie et al., 2021 [46]
Etheostoma cragini*	Arkansas darter	CSU_Ecrag_1.0	GCA_013103735.1	Reid et al., 2021 [47]
Gadus morhua*	Atlantic cod	gadMor3.0	GCA_902167405.1	Tørresen et al., 2017 [48]
Haliotis rufescens*	Red abalone	xgHalRufe1.0.p	GCA_023055435.1	Griffiths et al., 2022 [49]
Hippoglossus hippoglossus*	Atlantic halibut	fHipHip1.pri	GCA_009819705.1	Einfeldt et al., 2021 [50]
Hippoglossus stenolepis*	Pacific halibut	HSTE1.2	GCA_022539355.2	Jasonowicz et al., 2022 [51]
Homarus americanus*	American lobster	GMGI_Hamer_2.0	GCA_018991925.1	Polinski et al., 2021 [52]
Ictalurus furcatus**	Blue catfish	Billie_1.0	GCA_023375685.2	Waldbieser et al., 2023 [40]
Ictalurus punctatus*	Channel catfish	IpCoco_1.2	GCA_001660625.2	Liu et al., 2016 [39]
Ictalurus punctatus**	Channel catfish	Coco_2.0	GCA_001660625.3	Waldbieser et al., 2023 [40]
Lampris incognitus**	Smalleye Pacific opah	fLamInc1.hap2	GCA_029633865.1	Rhie et al., 2021 [46]
Lepisosteus oculatus*	Spotted gar	LepOcu1	GCA_040954835.1	Braasch et al., 2016 [53]
Magallana gigas*	Pacific oyster	cgigas_uk_roslin_v1	GCA_902806645.1	Peñaloza et al., 2021 [32]
Magallana gigas**	Pacific oyster	xbMagGiga1.1	GCA_963853765.1	Mrowicki et al., 2024 [33]
Mercenaria mercenaria*	Hard Clam/Northern quahog	ASM1480567v1.1	GCA_014805675.2	Song et al., 2021 [54]
Mercenaria mercenaria**	Hard Clam/Northern quahog	MADL_Memer_1	GCA_021730395.1	Farhat et al. 2022 [55]
Micropterus salmoides*	Largemouth bass	ASM1485139v1	GCA_014851395.1	Sun et al., 2021 [56]
Misgurnus anguillicaudatus**	Pond loach	HAU_Mang_1.0	GCA_027580225.1	Sun et al., 2023 [57]
Morone saxatilis*	Striped bass	NCSU_SB_2.0	GCA_004916995.1	Not published. NCSU, Raleigh, NC
Mugil cephalus*	Flathead mullet/Striped mullet	CIBA_Mcephalus_1.1	GCA_022458985.1	Shekhar et al., 2022 [58]
Oncorhynchus gorbuscha*	Pink salmon	OgorEven_v1.0	GCA_021184085.1	Christensen et al., 2021 [59]
Oncorhynchus keta*	Chum salmon	Oket_V1	GCA_012931545.1	Rondeau et al., 2021 [60]
Oncorhynchus keta**	Chum salmon	Oket_V2	GCA_023373465.1	Rondeau et al., 2023 [61]
Oncorhynchus kisutch*	Coho salmon	Okis_V2	GCA_002021735.2	Kim et al., 2016 [62]
Oncorhynchus mykiss*	Rainbow trout	USDA_OmyKA_1.1	GCA_013265735.3	Gao et al., 2021 [38]
Oncorhynchus nerka*	Sockeye salmon	Oner_1.0	GCA_006149115.2	Christensen et al., 2020 [63]
Oncorhynchus nerka**	Sockeye salmon	Oner_Uvic_2.0	GCA_034236695.1	Christensen et al., 2020 [63]
Oncorhynchus tshawytscha*	Chinook salmon	Otsh_v2.0	GCA_018296145.1	Christensen et al., 2018 [64]
Oreochromis niloticus*	Nile tilapia	O_niloticus_UMD_NMBU	GCA_001858045.3	Conte et al., 2019 [65]
Ostrea edulis**	European oyster	xbOstEdul1.1	GCA_947568905.1	Gundappa et al., 2022 [66]
Penaeus monodon*	Giant tiger prawn	NSTDA_Pmon_1	GCA_015228065.1	Van Quyen et al., 2020 [67]
Penaeus vannamei*	Pacific white shrimp	ASM378908v1	GCA_003789085.1	Zhang et al., 2019 [68]
Perca flavescens*	Yellow perch	PFLA_1.0	GCA_004354835.1	Feron et al., 2020 [69]
Procambarus clarkii*	Red swamp crawfish	ASM2042438v2	GCA_020424385.2	Xu et al., 2021 [70]
Ruditapes philippinarum**	Manila clam	ASM2657151v2	GCA_026571515.2	Xu et al., 2022 [71]
Salmo salar*	Atlantic salmon (European)	Ssal_v3.1	GCA_905237065.2	Lien et al., 2016 [72]
*Salmo salar*	Atlantic salmon (N. American)	USDA_NASsal_1.1	GCA_021399835.1	Gao et al., 2023 [35]
Salvelinus fontinalis**	Brook trout	ASM2944872v1	GCA_029448725.1	Pasquier et al., 2016 [73]
Seriola dumerili*	Greater amberjack	Sdu_1.0	GCA_002260705.1	Araki et al., 2018 [74]
Seriola lalandi dorsalis*	California yellowtail	Sedor1	GCA_002814215.1	Purcell et al., 2018 [75]
Syngnathus scovelli**	Gulf pipefish	RoL_Ssco_1.1	GCA_024217435.4	Ramesh et al., 2023 [76]
Thunnus albacares**	Yellowfin tuna	fThuAlb1.1	GCA_914725855.1	Ciezarek et al., 2016 [77]
*Trachinotus carolinus*	Florida pompano	FAU_TrCaro_1	GCA_040938265.1	Not published. FAU, Boca Raton, FL
Xiphias gladius*	Swordfish	ASM1685928v1	GCA_016859285.1	Wu et al., 2021 [78]

^*^Genome is included in current AquaMine v1.2

^**^Genome planned for next AquaMine release

Genome-enabled breeding going forward will likely utilize multiple types of genomic variation, including single nucleotide polymorphisms (SNPs) and structural variants (SVs), alongside gene annotation data to understand the heritable basis of production traits and to identify causative genome variants. Further, in the future this will be greatly facilitated by rapid and accurate AI/ML approaches. With the rapid and significant cost reduction of next-generation sequencing, low-coverage whole-genome sequencing followed by genotype imputation is becoming a cost-effective alternative to SNP array genotyping [79]. However, the production and use of SNP genotyping arrays will continue, and development of other cost-effective genotyping technologies is still necessary to comprehensively dissect genetic differences between lines, breeds, and populations of aquaculture species. An example of such advancements can be found in rainbow trout aquaculture, where new lines have been sequenced [38, 80], SVs have been identified in breeding populations [81], and the sex-determining gene was included in the Y chromosome sequence [38]. Maintaining SVs may also have important impacts withing breeding programs, for example, depletion of SVs has been identified among inbred lines of eastern oyster when compared to wild-type, suggesting that selective breeding can influence SVs [82]. This extensive library of data is foundational for breeding programs given the tremendous diversity of genetic architecture within and between populations of aquaculture species. Collectively these data enhance G2P assessments and increase precision of individual performance prediction within a breeding population.

One avenue for breeding is the identification of functional variants for specific selection of targets and traits. Genome-wide association studies (GWAS) are now commonplace [6, 83–93] and genome editing technologies, like CRISPR, can be used to validate gene function through genetic engineering [94–103]. These approaches have been used to produce distinct phenotypes [97, 104, 105], such as improved growth [99, 102, 106–108], disease resistance [99, 109], and sterility [97, 110, 111]. The emerging use of CRISPR genome editing technology in aquaculture will expand capabilities to identify functional variants helping to improve aquaculture production traits [112]. Evolving regulatory frameworks are facilitating the use of gene editing technologies in food animals in many countries, including the U.S. [113, 114]. The acceptance, implementation, and regulation of gene editing technologies in food production varies widely [115–117] and will likely continue to evolve as data accumulates on the health and safety of end products.

A different breeding approach is required for traits with polygenic structure, as no singular gene contributes a significant amount to variation of the trait. In such cases, pangenomic analyses that incorporate additional forms of genomic variation may identify networks that explain phenotypic variance or increase accuracy in estimating genetic relatedness, which drive increased effectiveness of GS. A pangenome is the set of core and dispensable genes within a species, and pangenomic variation is often structural, which has not yet become widely utilized in GWAS analyses [118]. High-quality genomes will benefit pangenomic analyses broadly through SV identification and give researchers the ability to interrogate SVs for phenotypic associations [119–122], species hybridization [40], and also give breeders the ability to discover, preserve, and utilize all genomic diversity within a species or line [82]. Approaches incorporating AI/ML techniques will also greatly enhance analysis and incorporation of pangenome data.

There is considerable variation in the spectrum of genome resources currently available for U.S. aquaculture species. While a number of established species have high-quality genome assemblies that have enabled advanced GS and genome-enabled breeding approaches to be utilized, other newly cultured species may be earlier in their development and at the initial stages of generating a reference genome (Table 1). High-quality genomes are better suited for functional annotation which further increases their applicability. Ongoing efforts are currently in place to create functional annotations for aquaculture species. In Europe, the AQUA-FAANG (Functional Annotation of ANimal Genomes)^2^ project aims to provide functional annotation information (e.g., quantify chromatin accessibility via assay for transposase-accessible chromatin with sequencing, or ATAC-seq) for the six aquaculture species with major industries in the European Union [123] including rainbow trout, Atlantic salmon, and common carp (*Cyprinus carpio)*. In the U.S., the FAANG project has included aquaculture species, and at present the primary focus is on rainbow trout [124]. It is important to note that genomic resources and tools are not confined to specific geographic regions and therefore advancements in this area, such as those made through international FAANG projects, can benefit aquaculture globally and should be shared and leveraged to best achieve sweeping progress in aquaculture.

The broad variation among species highlights the number of diverse species cultured in the U.S. aquaculture industry, and new species are added to the aquaculture portfolio every year [125–129]. Thus, while high-quality genome assembly is a current leading edge of aquaculture genomics, it must be acknowledged and embraced that the process of developing and implementing genomic resources in U.S. aquaculture will be a long road that will continue to include reference genome and genotyping tool production, with many species needing to start at the simplest of genome resource development. Information on the established broodstocks for U.S. aquaculture species maintained by public institutions (i.e., not for profit) can be found in Table 2 in addition to information for some broodstocks maintained by organizations supported by private or public–private partnerships.

**Table 2:** Broodstocks of U.S. aquaculture species maintained and available through public programs (i.e., universities, government) for food production. Information for private programs and/or private–public partnerships that maintain broodfish are indicated with an asterisk (*)

Species	Broodstock(s)
***Established***	
Channel catfish	Delta Select broodstock are produced via genomic selection at the USDA ARS Warmwater Aquaculture Research Unit, Stoneville, MS
Blue catfish	Delta Elite broodstock are produced via selection at the USDA ARS Warmwater Aquaculture Research Unit, Stoneville, MS
Atlantic salmon (North. American)	Broodstock are produced via genomic selection at the USDA ARS National Cold Water Marine Aquaculture Center, Franklin, ME
Rainbow trout	Broodstock are produced via genomic selection at the USDA ARS National Center for Cool and Cold Water Aquaculture, Leetown, WV and at the USDA ARS Small Grains and Potato Germplasm Research Unit, Aberdeen, ID
Nile tilapia	The Genetically Improved Farmed Tilapia (GIFT) strain is available worldwide
Striped bass	Domestic broodfish are produced via mass selection at the North Carolina State University Pamlico Aquaculture Field Laboratory (NCSU PAFL), Aurora, NC
White bass	Broodstock are produced via family-breeding at the USDA ARS Harry K Dupree Stuttgart National Aquaculture Research Center, Stuttgart, AR
Pacific oyster	Broodstock are produced via family-breeding at the USDA ARS Pacific Shellfish Research Unit, Newport, OR
Eastern oyster	Region-specific broodstock are produced via family-breeding at the USDA ARS National Coldwater Marine Aquaculture Center, Kingston, RI (New England); via mass selection, rotational line crossing, and genomic selection at the Rutgers University Haskin Shellfish Research Laboratory, Port Norris, NJ (Delaware Bay); via family-breeding/genomic selection at the Virginia Institute of Marine Science Aquaculture, Genetics, and Breeding Technology Center (VIMS ABC), Gloucester Point, VA (Chesapeake Bay); and via mass selection at the University of North Carolina Wilmington (UNCW), Wilmington, NC
California yellowtail	*Wild-caught and F1 domestic *Seriola dorsalis* broodstock are held and produced by Hubbs-SeaWorld Research Institute (HSWRI), San Diego, CA
Yellow perch	Genetically improved broodfish are produced via mark-assisted selection and distributed to aquaculture industry by the Ohio State University Center for Aquaculture Research and Development, Piketon, OH
***Newly-Cultured***	
Sablefish	A population consisting of wild-caught female broodstock, F1 male broodstock, and F1 neomale broodstock (used to generate all-female aquaculture populations) is held by NOAA Fisheries Northwest Fisheries Science Center (NWFSC) Manchester Research Station, Port Orchard, WA
Green abalone	*Broodstock held by The Cultured Abalone Farm, Goleta, CA
Largemouth bass	Broodstock are produced by selective breeding at the Ohio State University Center for Aquaculture Research and Development, Piketon, OH
Longfin yellowtail	*Wild-caught *Seriola rivoliana* broodstock are held by Blue Ocean Mariculture, Kailua-Kona, HI
Red drum	A seedstock development program is beginning at the new USDA ARS National Warm Water Marine Research Unit, Ft. Pierce, FL
Pompano	A seedstock development program is beginning at the new USDA ARS National Warm Water Marine Research Unit, Ft. Pierce, FL
Kumamoto oyster	A population is propagated by the USDA ARS Pacific Shellfish Research Unit in Newport, OR. No selection occurs on this species currently, captive spawns are aimed to maximize the retention of genetic diversity and limit inbreeding accumulation
***Protected/Other***	
White abalone	Broodstock held by partners of the White Abalone Captive Breeding Program, including: University of California Davis Bodega Marine Laboratory, Bodega, CA; NOAA Fisheries Southwest Fisheries Science Center, La Jolla, CA; Aquarium of the Pacific, Long Beach, CA; Santa Barbara Museum of Natural History Sea Center, Santa Barbara, CA; and Cabrillo Marine Aquarium, San Pedro, CA
Black abalone	Broodstock held by the NOAA Fisheries Southwest Fisheries Science Center, La Jolla, CA and University of California Davis Bodega Marine Laboratory, Bodega Bay, CA
Red abalone	Broodstock held by the NOAA Fisheries Southwest Fisheries Science Center, La Jolla, CA *Broodstock are also held by the Monterey Abalone Company, Monterey, CA and The Cultured Abalone Farm, Goleta, CA
Olympia oyster	Broodstock are held at the Kenneth K. Chew Center for Shellfish Research and Restoration conservation hatchery housed at the NOAA Fisheries Northwest Fisheries Science Center (NWFSC) Manchester Research Station, Port Orchard, WA and jointly supported by the Puget Sound Restoration Fund (PSRF) nonprofit
Pinto abalone	A hatchery is housed at the NOAA Fisheries Northwest Fisheries Science Center's Mukilteo Research Station in Mukilteo, WA

### Aim 2: Integrating multi-omics data to enhance analysis of production and performance traits

**Fig. 2:**
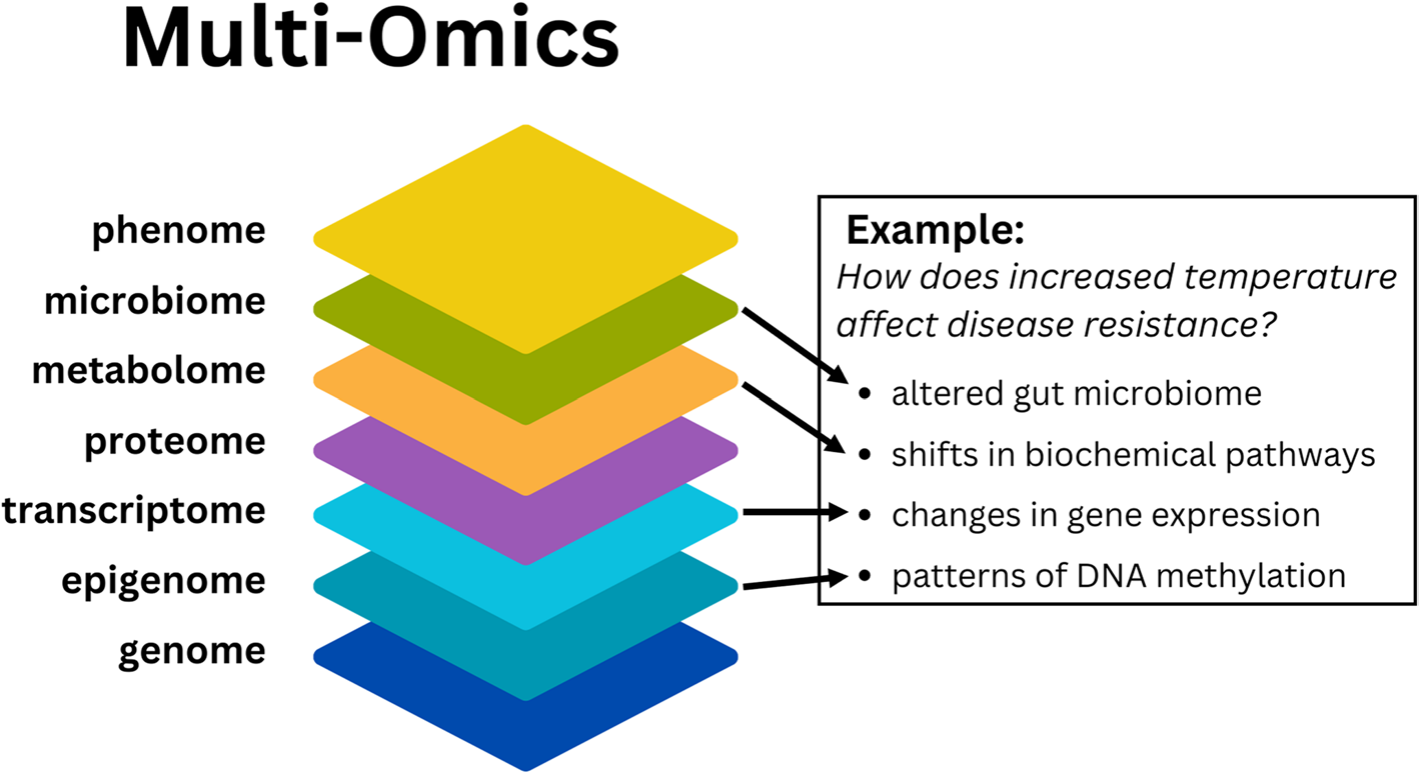
Major omics areas of focus from the phenome to genome and an example of how they can integrate to answer research questions

While genetic information is encoded in the genome sequences, the realization of performance and production traits are regulated at multiple levels between the genotype, observed phenotype, and the environment. Regarding these levels as separate scientific disciplines, the primary omics areas used to decipher molecular components and cellular processes in aquaculture organisms are genomics, epigenomics, transcriptomics, proteomics, microbiomics, and metagenomics. A single omics layer provides a massively parallel set of data for a given component. As examples: Genomics is the study of the complete set of DNA sequences within an organism, tissue, or cell that enables investigations of genetic variation and heredity. Epigenomics is the study of non-mutational modifications to DNA and RNA that provide information on gene regulation, chromatin structure, chemical modifications, and epigenetic inheritance. Transcriptomics is the study of RNA transcripts that allows for quantification of gene expression patterns, alternative splicing, and regulatory mechanisms, including that of non-coding RNA expression. Proteomics is the study of the entire set of proteins that explores protein structure, function, and interactions. Metabolomics is the study of the metabolites or chemical signatures that provide information on cellular processes, pathways, and metabolic regulation. Microbiomics is the study of the composition and function of microbial communities associated with the host and/or their environment; and metagenomics is the study of all genetic material collected from a sample that can include DNA from any organism present in the environment beyond the host organism (Fig. 2). The incorporation of two or more of these layers in an analysis is a “multi-omics” approach (also referred to as poly-omics, pan-omics, trans-omics, integrative omics, vertical omics, and systems omics) [29].

Many transcriptome analyses of aquaculture species have demonstrated both the complexity and power of transcriptomics for decoding phenotypes. For instance, transcriptome analyses led to the identification of genes related to low oxygen response in channel catfish [130, 131] and patterns of gene expression related to *Flavobacterium columnare* susceptibility and resistance in white bass (*Morone chrysops*) and hybrid striped bass (*M. chrysops* x *M. saxatilis*) [132]. Disease challenges can induce expression of a large numbers of genes, and further comparisons of resistant and susceptible lines (e.g., distinct populations) provide insights into important genes underlying resistance (e.g., catfish [133, 134] and eastern oyster [135–137]). Studies of post-transcriptional processing, such as RNA splicing and poly-adenylation have also led to powerful discoveries, especially in the context of the environmental impact on genetic regulation (i.e., gene-by-environment, GxE interaction), presumably through induction of environment-sensitive promoters and enhancers, and through epigenetic regulation. For instance, bacterial infection [138, 139] and heat stress [140] drastically increased the level of alternative splicing, and alternative polyadenylation [141] in channel catfish. Similarly, an Iso-Seq (isoform sequencing) study used to improve the rainbow trout genome annotation identified alternative splicing associated with economically important phenotypes, including resistance to bacterial cold-water disease and stress tolerance [14, 142]. Analysis of transcriptomic, proteomic, and/or metabolomic data that incorporate AI/ML algorithms have been applied to determine omic signatures of growth and body size (e.g., hybrid striped bass [143]), predict egg quality (e.g., striped bass, *Morone saxatilis*, [144, 145]), identify responses to chemical exposure or developmental state (e.g., striped bass [146] and white perch, *Morone americana* [147]), predict disease resistance (e.g., common carp [148]), and detect seafood traceability signatures (e.g., wild and aquacultured shrimp [149]). Further, advanced sequencing technologies now allow for transcriptomic resolution at the single-cell and spatial levels, the findings of which are anticipated to be of similarly high value and impact to aquaculture [150–154]. Continued investment in high-resolution functional annotations of reference genomes is crucial for maximizing the utility of transcriptomics and other omics-based tools.

In the last decade, huge progress has been made in understanding the impact of epigenomic regulation on phenotypes and related research strongly supports the contribution of epigenetic modifications to additional layers of variation that can be targeted to improve production-relevant traits related to reproduction, health, growth, and nutrition of agricultural animals [155–158]. Although such studies are still at the infancy stage with aquaculture species, several have demonstrated the epigenetic regulation of important production traits. For instance, an epigenetically marked locus was identified to be associated with sex determination in channel catfish [159] and allelic expression of *hydin-1*, the candidate master sex determination gene of channel catfish, was found to be regulated by DNA methylation [160]. Gong et al. (2023) [161] found that spatial regulation is crucial for the expression of the yellow catfish (*Pelteobagrus fulvidraco*) master sex determination gene, *pfpdz1*. Differentially methylated regions have been identified on the promoter regions of cell signaling and embryonic development genes in Atlantic salmon fed diets of differing micronutrient concentrations [162], and associated with high- and sub-fertility in male striped bass [163], polyploidy and muscle atrophy in rainbow trout [164], and the immune response of eastern oyster following infection with the protistan parasite *Perkinsus marinus* [165]. In addition to the identification of core biomarkers that are opportunities for epigenetic selection, leveraging of environmental manipulation and epigenetic memory to improve phenotype can be directly employed to enhance aquaculture [157]. In some cases, improved performance has been realized in the absence of characterizing the precise epigenetic mechanism (e.g., Olympia oyster, *Ostrea lurida* [166]; Pacific geoduck, *Panopea generosa* [167]), whereas in other studies the functional role of DNA methylation in contributing carryover effects have been described (e.g., Pacific geoduck [168]). There has also been valuable work regarding the degree and genomic mechanism by which genetic and epigenetic variation are associated, providing insight into ecotoxicological pollution burdens [169] and how selection and manipulation can be used in the future to improve phenotypes (e.g., Olympia oyster [170]). The overall roles and the diversity of epigenetic mechanisms across major taxa and insights into their potential applications in the culture of aquatic animals are vast and have been recently reviewed [155–158].

Microbiomics and metagenomics studies in aquaculture have largely focused on two areas: (1) gut/intestinal microbiota with the aim of examining fish health, welfare, digestion, and immune response, and (2) environmental samples, with the aim of improving fish health and production efficiency by targeting environment-associated problems in aquaculture systems. The gut microbiome is crucial in maintaining good health, homeostasis, and metabolism by acting as a barrier against harmful bacteria and the production of vital vitamins and metabolites. Composition of the gut microbiome is considered an "intermediate phenotype" that results from host genetics and environmental influences [171], and is considered part of the whole genome, or "hologenome.” As the host's genetics significantly shape the gut microbiome [172], the term "microbiability" has been introduced to describe a concept similar to heritability. Awany et al. (2019) [173] reviewed the interaction between the host and the microbiome and its impact on the host's physical characteristics broadly. Since then, studies have demonstrated the association between rainbow trout gut microbiome, growth, and muscle percentage [174–176], and shellfish microbiome differences that influence growth [177] and survival against viral disease [178].

Investigating the microbial organisms present in aquaculture environments provides information on the diversity and composition of both beneficial and harmful microorganisms that can serve as indicators of environmental health, inform disease prevention and management strategies, influence nutrient cycling and waste management, and help optimize feed composition and regimes. Shotgun metagenomics and, more commonly, amplicon sequencing, have been utilized for microbiome profiling in various aquaculture production systems (RAS, or recirculating aquaculture systems; biological floc, or biofloc; and ponds) and for conservation purposes in areas with coexisting native fish. These studies have focused on identifying optimal sample preparation/sequencing methods [26, 179], evaluating the effects of different environmental parameters/conditions [180], and characterizing communities associated with different aquaculture systems [181–183]. Environmental DNA (eDNA) coupled with amplicon sequencing have been used to monitor abundances of fish pathogens [184] and invasive fish species [185]. Additionally, metagenomics coupled with metaproteomics has been used to describe the relative functional role of the microbial community in a bivalve hatchery under different conditions [186].

The integrative multi-omics approach of simultaneously analyzing multiple omics layers of a system, ranging from single cell to whole organism, provides a more comprehensive, or holistic, understanding of intricate molecular networks that govern biological processes. In agriculture, multi-omics approaches can enhance breeding by providing greater insight into the genetic and molecular basis of desirable traits and subsequently improving selection accuracy [187, 188]. Multi-omics approaches can similarly improve resource optimization by identifying key molecular factors that influence traits such as growth, disease resistance, and stress tolerance. There are many considerations for the integration of multi-omics data and interpretation of results as data generation and quantification methods differ between omics fields. These considerations include, but are not limited to, data standardization, cross-platform compatibility, data and metadata handling and completeness (e.g., missing, imbalanced, etc.), analysis (i.e., reduction) methods, data reporting, and biological validation approaches. Cross-platform compatibility is a significant challenge with ML analyses of multi-omics data, principally due to differences in data reporting and standardization which, if unaccounted for, can lead to biased statistical outputs [189–191]. Workflows and applications to integrate multi-omics data for analysis and/or visualization are continuously being developed as the number of technologies that facilitate the collection of these data and the accessibility thereof increase. Examples include: MOMIC [192], OmicsSuite [193], Bioconductor MultiAssayExperiment [194], IDARE2 (Integrated DAtanodes of REgulation) and Cytoscape [195, 196], multi-omics [197], and those listed in Chakraborty et al. (2022) [187]. Additionally, analytical pipelines based in AI/ML are continuously being developed to integrate these data types and address cross-platform issues as the models employed are not restricted in the same manner as traditional statistical approaches [198]. The suitability of these and other multi-omics integration tools must be evaluated case-by-case, as many have been developed using data generated from plant or model animal species and therefore may not sufficiently account for nuances of some aquaculture species (e.g., whole genome duplication). Further, the aquatic environment and effects thereof present an opportunity to incorporate additional data that is different from plants and terrestrial organisms. As such, developing tools specifically suited to aquaculture data and expanding our knowledge of them is a much-needed critical step forward.

The integration of multi-omics approaches in agricultural sciences is a paradigm shift, offering unprecedented insights into the complexity of biological systems. The holistic perspective of distilling these complex interactions into quantifiable factors associated with phenotypic variation advances basic science and holds tremendous promise for practical applications, such as breeding, genome editing, and improved selection accuracy using genome-enabled selection [199]. Establishing standards for data integration and the development of comprehensive workflows will greatly contribute to the robustness and reliability of multi-omics studies, further positioning them as indispensable tools in the pursuit of sustainable and efficient food production.

### Aim 3: Developing resources for the collection and integration of phenomics data

Phenomics is the study of observable traits that contribute to the expression of a phenotype [200]. Phenotypic variation is equally, if not more, important to genetic selection programs than genetic and genomic data [199]. Recent advances in sequencing technologies have allowed the application of multiple genome-enabled selection strategies to be at the fingertips and keyboards of aquaculture scientists. While cost and technical laboratory challenges of genetic data production remain hurdles, they no longer represent the major barrier for advancing animal breeding. The most significant barrier is currently high-throughput phenotyping and the creation of multi-trait phenomes that can be quantitatively assessed [201].

Zebrafish (*Danio rerio*) lead all finfish in phenomics data resources (reviewed in Fuentes et al., 2018 [202]), and this species represents the exception within aquatic species rather than the rule. Zebrafish are a model species for biomedical researchers due to the ease of husbandry, short generation time, and fewer genome duplication events than other fishes. Understanding individual gene action to phenome expression is laborious, expensive, and development rates are dependent on the generation time of the animal (i.e., age to sexual maturation) and ease of gene manipulation. Some aquaculture species lend themselves to this model (e.g., tilapia, *Oreochromis spp.*), while others have longer generation times (e.g., salmonids, catfish) or are challenging to introduce gene modifications (e.g., shellfish). Thus, the development of phenomics datasets via gene knockout or gene modification is not likely to be widely applied within aquaculture species at scale until the discipline matures and technologies are available that can reliably and efficiently modify organisms at various life cycle stages (e.g., eggs/zygote).

An area where phenomics has the potential to significantly influence aquaculture is via automated, high-throughput data capture. Advances in this area are occurring through the combination of imaging and/or sensor technologies with AI/ML models being able to identify diseased individuals in intensive aquaculture settings [203], count individuals within an enclosure [204], produce real-time body size data [205], estimate biomass changes [206], signal an alert system based on detected mortality events [207], classify shape of an organism (e.g., oysters, [208]), determine feed conversion efficiency [209], predict readiness to spawn [210], identify shellfish gonads (e.g., Pacific oyster, [211]), monitor interactions between marine aquaculture animals and wild populations [177], and estimate moisture, glycogen, and ploidy in shellfish (e.g., eastern oyster, [212]), among numerous other applications [213]. These advances set the stage for production-level questions to be answered for entire lots of animals with potentially lower labor costs and fewer data collection errors (e.g., reduced subjectivity). Additionally, post-harvest traits of quality, such as seafood product freshness, may be assessed via e-systems (e.g., e-eye, e-nose, e-tongue) that quantitatively assess quality and represent a leap forward in data automation for product processing from farm to market [214, 215].

Multiple challenges exist for integrating phenomics data into aquaculture programs. Aquaculture occurs in environments harsh on equipment, and development of sensors and cameras that are able to withstand aquatic environments and extreme weather events is needed. Efforts to install infrastructure and implement strategies to obtain phenomics data from these sensors and cameras (e.g., via internet connection, collection of memory cards) will also have to be emphasized, as this is similarly challenging in aquatic environments. Moreover, accessible software capable of processing raw data into tangible datasets that can be used for phenomics trait production must also be developed. The scales and methods of aquaculture operations vary widely, with large programs (e.g., salmon and trout) being more likely to adopt and afford these high-throughput systems. Accessibility and democratization across program scale and affordability for smaller scale programs (e.g., shellfish, newly cultured finfish species, etc.) will be necessary for aquaculture as a discipline to reap the benefits of phenomics. Initial efforts in generating genomic and omics resources for newly cultured species, and others at similar stages of industry development, should focus on developing high-quality reference assemblies that will anchor the layers of omics and phenomics data.

While production may benefit from population or group level measurements, breeding efforts require integration of individual phenotypic variation with genomic data. This is especially challenging in aquaculture environments where animal tagging is difficult and/or expensive to conduct and maintain. PIT (passive integrated transponder) tags can be implanted, yet to date there are limited records of combining tag readers with non-lethal automated data collection [216–218]. Developing technology that can simultaneously identify individuals and collect phenotypic data is a frontier for advancing aquaculture phenomics (e.g., Babu et al., 2022 [204]). The rapid advancements that are occurring in genomics and multi-omics cannot be fully exploited without similar advancement in and integration with phenomics.

### Aim 4: Creating pathways for applying and integrating genomics information across animal industries

The integration and application of genomics data across animal agriculture sectors plays a pivotal role in driving impactful changes and improvements in these industries. Specifically, sharing omics resources and strategies used in diverse animal industries facilitates the increased leveraging of these data and tools to benefit myriad groups, including academic and industry researchers, Extension specialists, producers, and other stakeholders. For example, resources for genome data mining and visualization such as AquaMine (http://aquamine.org; Elsik Lab, University of Missouri) and AgAnimalGenomes [219] enable the exploration of genomic and omics data within and between animal production sectors without requiring advanced training. These and similar data exploration tools lend themselves to powerful comparisons, which, among many other potential findings, can help identify conserved pathways between species that can be exploited to enhance production-relevant traits. The field of aquaculture is inherently and uniquely positioned to collaborate with neighboring fields like fisheries management, molecular ecology, and conservation genomics given the overlap of study species and experimental approaches used to answer fundamental questions. This interdisciplinary nature of aquaculture research can and should be expanded to other agriculture sectors as we collectively work towards supporting global food security.

The application and integration of data-based tools and resources within and across animal industries relies on the generation, maintenance, and provision of high-quality omics data and detailed metadata that meets agreed-upon standards of interpretability and availability. Several initiatives have developed such standards for genomic data and analysis, including the MIxS (Minimum Information about any (x) Sequence) checklist for metadata produced by the Genomics Standards Consortium (GSC) [220]; recommendations for analysis standards from the Earth BioGenome Project [221]; and published guidelines for protocols and data standards for multiple omics project types published by the ENCODE Consortium (Encyclopedia of DNA Elements) [222, 223]. Broader guidelines for scientific data reporting, such as FAIR Principles (Findable, Accessible, Interoperable, Reusable) [224], and tools that improve FAIRness of resources, such the Bioschemas effort to embed distinct markup into published data (e.g., genes, chemicals, proteins), workflows, and training programs [225–228], have also been developed and should be considered and incorporated into the determined set of data and metadata standards. The current aims of the latest iteration of the NRSP8,^3^ Building Applied Genomic Capacity for Animal Industries, echo these sentiments.

**Fig. 3:**
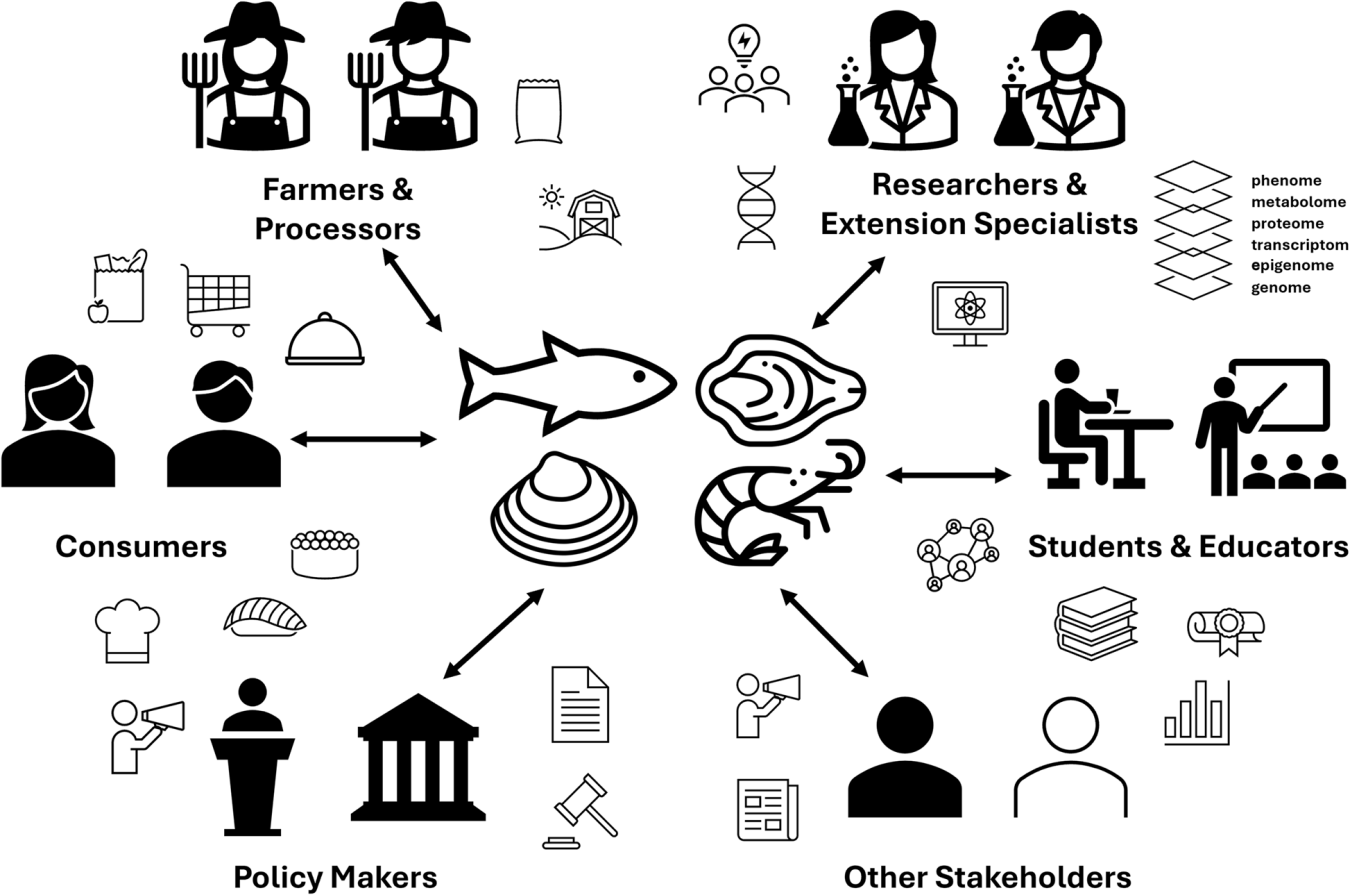
Input from multiple groups impacts the success of aquaculture production from lab to farm to plate

Further, consensus on joint investment and participation of researchers, Extension specialists, agency personnel, and other stakeholders is necessary to best facilitate the sharing of feedback, ideas, and innovations. Funding organizations, institutions, and individuals with an interest in animal and plant production should prioritize hosting and/or participating in forums for feedback, discussion panels, workshops, etc., such that persons and groups invested in U.S. agriculture can collaborate. As examples, USDA NIFA accepts investigator-initiated proposals for conferences and workshops year round, and the Aquaculture Information Exchange,^4^ an online forum to facilitate discussion between members of the public and private sectors with interests in U.S. aquaculture, was launched in 2023 by Virginia Sea Grant, with support from the National Oceanic and Atmospheric Administration (NOAA) National Sea Grant Office, NOAA Fisheries Office of Aquaculture, USDA Agricultural Research Service (ARS), and USDA NIFA. The Plant and Animal Genome Conference (PAG) held annually in San Diego, California served as a meeting place for those involved in or affiliated with the initial NRSP8 NAGRP, where interaction between agriculture sectors was nurtured and facilitated. Consistency to this end (i.e., location) provided an ease of knowing at least one avenue for involvement of academic and government researchers, students, industry professionals, and other contributors (Fig. 3). However, a single annual meeting is not sufficient for establishing long-term, productive relationships within and among animal sectors, especially when the strengths come from the diversity of species groups, analytical approaches, and stakeholders from private sector (industry), government, and academia. These interactions are invaluable for integrating new insights, technologies, and analysis types into agricultural practices that have real-world impacts. As such, support and energy behind collaborative efforts must continue, if not increase, with the ever-advancing omics- and data-intensive technologies to optimally and most efficiently leverage these tools toward the improvement and strengthening of U.S. agriculture.

### Aim 5: Providing training, extension, and outreach to support the application of genome to phenome

“Genome to phenome” broadly refers to the linkage between the genetic blueprint (genome) and the observable traits of an organism (phenotypes, or phenome [3, 229, 230]. Research with this focus is designed to further our understanding of how molecular components and environmental factors influence complex phenotypes and is highly relevant to agriculture. Applying G2P research across agriculture sectors is critical to addressing food security challenges that continue to escalate with the growing global population, unpredictable and more frequent environmental impacts (e.g., hurricanes, floods, droughts, etc.), and biological events (e.g., emergent animal disease outbreaks, COVID-19 pandemic). Consortiums such as the Agricultural Genome to Phenome Initiative (AG2PI; https://www.ag2pi.org/) have been developed to address challenges and advance G2P research across agriculture sectors, including data sharing and integration, and the development of tools to evaluate and measure phenotypes [229, 230].

The establishment and continued support of programs that train early-career professionals with the requisite skills to navigate the complexities of genomics in agriculture and facilitate their integration into the workforce is paramount to achieving the ultimate goals of G2P research and delivering nutritious aquatic animal products to a growing global population. Specifically, this workforce must be efficacious in conducting and reporting scientific research, developing and commercializing tools, and communicating outcomes to farmers, consumers, legislators, and future generations. Educational programs that provide a foundational understanding of genomics, phenomics, and their integration into animal breeding and management practices must be developed and made broadly available at general and advanced levels to support the range of workforce personnel needs. An example is the interdisciplinary Genomics, Phenomics, and Bioinformatics (GPB) graduate (PhD and MS) program at North Dakota State University, which is described^5^ as:

*“The program is designed to provide students the necessary skills and intellectual background to work cooperatively with others in a research area that take a systems-wide approach to the study of the organization of life and expression and regulation of genes in an organism. Students in the program will perform advanced study, training and research in areas that focus on functional genomics, high-throughput phenotyping, and computation analysis of genomic and phenomics data. Students will learn and master the multi-omics approaches for research in many frontiers. Exposure to modern techniques, instrumentation, computational and statistical methods will prepare the student for success in both industrial and academic careers."*

Another example of this effort is the recently-funded (2024) USDA NIFA project “Graduate Education in Livestock Phenomics and Quantitative Genomics” awarded to Texas A&M University (Research, Education, and Economics Information System, REEIS, accession no. 1031671) that aims to “(1) recruit, (2) develop, and (3) produce outstanding graduates in the animal production targeted expertise shortage area who will become the next generation of leaders in animal breeding equipped with expertise in phenomics and associated quantitative genomics methods.”

Internships, work-study, and similar programs should be designed by or in collaboration with industry to ensure that early-career professionals are gaining relevant hands-on experience and, if possible, direct training for future positions. This can include partnerships with industry, or other relevant groups, such that successful participants who wish to pursue a career in the field can be placed efficiently into the workforce. There are several examples of these programs for plant crops, such as the USDA NIFA Research and Extension Experience for Undergraduates (REEU) program for Phenomics Big Data Management at Washington State University.^6^ Similar programs should be established for animal/aquaculture production and include the spectrum of involved parties. For example, Extension personnel affiliated with U.S. Land-grant Colleges and Universities (1862, 1890, and 1994) and NOAA Sea Grant Programs are often the first line of communication with aquaculture producers who would like to apply genomics in their production systems. Therefore, inclusion of Extension personnel in research partnerships and training programs greatly expands opportunities for technology transfer to industry stakeholders as well as workforce development and professional relationship building.

Further, supporting the attendance and participation of early-career professionals at conferences, stakeholder meetings, workshops, etc. should also be emphasized as these opportunities allow for advanced and continued learning, sharing of ideas, and the development of professional relationships within and outside of each specific area or field of work/research.

The establishment and support of comprehensive training, Extension, and outreach programs are pivotal to achieving the goals of G2P research and to efficiently and effectively transfer developed technologies to the commercial aquaculture industry. By preparing a well-equipped workforce, fostering industry collaboration, and promoting continued professional development, we can enhance the productivity, sustainability, and resilience of agriculture to benefit both producers and consumers.

### Future research priorities & directions

One thrust of the 2023 Workshop for Aquaculture Genomics, Genetics, and Breeding was to identify priorities for the next decade and beyond based upon what is anticipated to become the cutting edge of aquaculture omics research in the U.S. and projections of what will be needed to utilize such research to help meet the needs of the global population. Here we outline these ideas as a suggested guide to where U.S. aquaculture genetics, genomics and breeding research resources should be directed in the years ahead. It must also be acknowledged that global research advancement and international collaboration will only benefit aquaculture omics research and is one of the most efficacious ways to produce formidable advancement.

#### Genome-enabled selection models and applications

The identification of functional genes has the potential to rapidly advance U.S. aquaculture and this will be greatly facilitated by the inclusion of AI/ML approaches in analysis pipelines [123, 148, 213]. Genes underlying reproductive sterility, growth, stress, and disease resistance provide commercial breeders with essential data to produce animal populations better adapted to production environments and systems [4, 231]. Creating sterile populations could significantly enhance the social license of aquaculture to operate by significantly reducing, if not functionally eliminating, the potential for introgression between captive and wild/natural populations. While identifying traits underlain by simple genetic architectures and individual genes (or small subsets of genes) will advance the aquaculture breeding field, relatively few traits will be classified as such. The phenotypes targeted through selection (i.e., breeding) programs are often quantitative, complex traits that are influenced by a large number of loci with small effect sizes [4].

For traits with polygenic architecture, the continued development of GS workflows (from genotyping to statistical analysis) is vital [11, 232–235]. These traits are best suited for classical quantitative genetic analysis, using genome-wide measures of relationship between individuals, rather than focusing on shared genetic variation from a subset of chromosomal segments. Genome-enabled selection programs will become more common throughout aquaculture as genotyping costs decrease, sample throughput increases, and the potential benefits of improved selection accuracy are realized into tangible production outcomes (i.e., more rapid genetic gain) [236]. How quickly U.S. aquaculture breeding programs adopt GS is predicated on the availability of trained staff, funding, and reliable, affordable tools to genotype and phenotype animals.

Cost reduction is especially important in aquaculture programs [237], as there are high numbers of breeding candidates in a given population and the market value of each individual is low relative to other livestock animal industries. Continued development of inexpensive, high-throughput tools for genomic/omics data generation is needed. Our understanding of how large (i.e., total number of loci assayed) a genotyping panel must be to maximize GS accuracy is changing as studies have found that only hundreds to thousands of loci are needed to achieve highest accuracy [83, 90, 238, 239]. Panel size reduction may be possible using imputation-based methods [11] and can be combined with pedigree information [232] and microhaplotype variation [233, 240] to further reduce panel sizes via increased statistical power. It is likely that geneticists will soon be able to routinely produce the genome-level data required to maximize the accuracy of GS models. However, the advancement of GS application will be constrained by the cost of genotyping and a lack of efficient, scalable, and cost-effective phenotyping methods for aquaculture species.

#### Pangenomics

The substantial diversity within and among cultured species and production methods sets aquaculture uniquely apart from other major animal production industries. This is a challenge in the limited portability of tools across aquaculture species, but an opportunity is also present as the genetic variation within aquaculture species is primarily untapped. In this vein, the continued advancement of pangenomics in aquatic species is important to discover, utilize, and preserve the genetic diversity available to breeding programs. Pangenomics also stands to introduce new types of genetic variation into GWAS analyses (e.g., SVs, gene deletions) that may influence trait expression. Pangenomic studies in cattle revealed that deletions were associated with breeds and subspecies delineations [241]; in plants, pangenomic studies have identified disease tolerance and yield associated genes [242]. Livestock and poultry have been at the forefront of the domestic animal pangenomics field, yet despite being in their early stages, multiple studies have already identified genetic variants associated with production traits that could not be quantified using singular genomic resources [243]. For pangenomics to impact aquaculture breeding, it is likely that bespoke pangenomes will be necessary for individual operations, given the decentralized model of hatchery production and limited direct link between programs compared to livestock and plants. The degree to which this is necessary will depend on many factors, but largely be driven by evolutionary relationships among broodstocks used by independent hatchery suppliers. Ultimately, quantifying pangenome variation allows for more comprehensive assessment of trait variation and increases the potential to identify functional regions and/or genes [244]. The opportunity of pangenomic research in aquaculture is enticing and necessary, as variation among ecotypes within species and across the multitude of closely related species will provide substantial fodder for trait and phenotypic dissection.

#### Phenomes (traits) of the future

Defining and identifying phenomes of interest for aquaculture is needed, as the ability to produce and integrate phenotype data on a production scale, rather than the production of omics data, is presently limiting aquaculture advancement [3]. Aquaculture has historically focused on growth, reproduction/sexual dimorphism, and feed conversion as selection traits. Rapidly changing environments, dietary (feed) components, and pests and pathogens in production environments are shifting selection targets toward resiliency phenotypes [245, 246]. In rainbow trout and Nile tilapia, resilience was estimated via a body weight phenotype across multiple temporal and spatial scales (production environments) with resiliency found to be heritable [247] with significant variance among lines [248]. These studies empirically demonstrate resilience can be applied in selective breeding to increase uniformity in the face of disturbance. In eastern oyster, production in lower salinity coastal environments is influenced by intensifying seasonal precipitation and freshwater diversions from major rivers for coastline stability, thus resilience defined by tolerance to low salinity stress is emerging as a breeding target with progress made on utilizing GS to advance this trait [84, 249, 250]. Disease resistance against viral [251–256], bacterial [257, 258], and parasitic pathogens [135, 252, 259] constitutes a core theme of resiliency phenotypes as well [5, 260].

To this end, defining resilience for each species and production environment is crucial. For example, shellfish reared in an intertidal environment may have a unique set of resilience traits that differ from resilience-conferring traits in a finfish program sited in the same waterbody. Within a single species, it is possible that suites of traits that define resilience phenomes vary based upon location (environmental characteristics) and/or production environment/system (e.g., open water net pen vs. recirculating aquaculture system). A common resilience trait among aquaculture species is the ability to survive and efficiently grow in dynamic conditions with the potential ability to respond to different stressors over time. Interestingly, resilient phenomes of multiple livestock and aquaculture species reared in varied agricultural systems have been found to share stress-associated pathways that contribute significantly to the phenotypes [261]. Therefore, resilience phenomes among species may be more similar than we currently hypothesize. Overall, identifying and developing the phenomes of the future will require significant effort. Effective methods are likely to be those which identify animals with high growth rate, high feed conversion efficiency, and enhanced resistance to disease and environmental stress in variable conditions.

While there is great promise of integrating multiple data streams into a composite phenome, advancements on specific traits can be made and should be prioritized. For example, producing value-added, healthier human food through targeting specific fatty acids in salmonids. Omega-3 long-chain polyunsaturated fatty acids (LC-PUFA) are essential for the health of both humans and fish, particularly for heart and brain function. However, aquaculture-produced salmon must consume approximately 2% of their diet from unsustainable sources of wild fish oil to maintain sufficient levels of LC-PUFA [171]. In this case, an individual trait such as LC-PUFA synthesis may benefit from phenomics approaches and reveal unexpected relationships that can be targeted to yield genetic gain.

#### Education, training, and program participation

Investment in education, training, and retention initiatives is imperative to the continued operation, future success, and fortification of U.S. agriculture, including aquaculture. Programs, such as those described above, will create a well-equipped workforce capable of advancing genomic/omics research and its applications, subsequently leading to improved agricultural productivity, sustainability, and food security. This includes establishing and maintaining programs to recruit students and interns for training as future educators, researchers, Extension personnel, and industry leaders. Strengthening collaborative ties between research, Extension, industry, and other stakeholders through the creation and utilization of centralized communication networks and information forums is vital to moving the aquaculture industry forward. Participation at any level is necessary and strongly encouraged, whether through securing or providing funding, establishing training-to-workforce pipelines, organizing or leading workshops and meetings, or contributing in other ways (e.g., Extension, outreach, public education). Collectively, these efforts ensure ongoing commitment and “buy-in” from all stakeholders, maximizing impact and promoting the continuous advancement of aquaculture and agriculture.

### Conclusion

The aquaculture genetics, genomics, and breeding community has made significant advancements over a short period, yet new and exciting challenges lie ahead. The next frontier in U.S. aquaculture production is to harness high-quality genome assemblies and integrate them with multi-omics and pangenomic approaches to distill the genetic variance associated with phenomes. However, it should not be overlooked that the expansion of U.S. aquaculture production via continual introduction of new species will require generation of novel genetic tools and the creation of robust phenotyping technologies. Unpredictable global events, such as those highlighted by the COVID-19 pandemic, have brought the importance of food security and food chain stability across the world into focus. The opportunity afforded by the U.S. aquaculture sector to help address food security is vast, and with continued development of genome-enabled breeding and genome editing strategies, omics integration, phenomics curation, and robust training, the future looks bright for the continued expansion of finfish, crustacean, and mollusk aquaculture in the United States.

Based on the present status of aquaculture genetics, genomics, and breeding research, and industry needs, the following areas should be prioritized to continue developing and strengthening the U.S. aquaculture sector:

1. Improvement of reference genome assemblies: Produce genome sequences of newly cultured aquaculture species and continue advancing the assembly and functional annotation of existing reference genomes including epigenome annotation, Genotype-Tissue Expression (GTEx), eQTL and epi-QTL (expression and epigenetic quantitative trait locus, respectively) analyses.
2. Advancement of pangenomics: Quantify and utilize all forms of genetic variation within and among species to advance G2P understanding and enhance breeding programs.
3. ​​Expansion of genome-enabled workflows: Develop cost-effective, scalable methods of selecting and utilizing genomic/omics data to increase selection accuracy and improve genome editing technologies to facilitate a greater functional understanding of G2P in aquaculture species. AI/ML will play a vital role in such workflows.
4. Defining resilient phenomes: Use genomic/omics data to identify and integrate both production-relevant traits (e.g., growth, disease resistance) and traits associated with environmental stress tolerance across varied culture environments and production systems.
5. Data integration and utilization: Adopt data and metadata standards to improve the accessibility and integration of multiple data types within and between species and to enhance applicable use of these data (e.g., comparative omics).
6. Education and training programs: Invest in initiatives to train a skilled workforce capable of advancing and applying genomic technologies in the aquaculture sector.

## Supplementary Information

Supplementary Material 1.

## Data Availability

No datasets were generated or analysed during the current study. The authors declare no competing interests.

## Footnotes

1 NRSP8 NAGRP Bioinformatics web page: https://www.animalgenome.org/bioinfo/community/mission

2 AQUA-FAANG website: https://www.aqua-faang.eu/about-us.html

3 2023-2028 NRSP8 project website: https://nimss.org/projects/view/18969

4 Aquaculture Information Exchange registration page: https://aquainfoexchange.org/register/

5 North Dakota State University GPB program: https://catalog.ndsu.edu/programs-study/graduate/genomics-bioinformatics/

6 USDA NIFA REEU for Phenomics Big Data Management: https://labs.wsu.edu/sankaran-phenomics/undergrad-research-exp/

## References

[1] Biga P, Rexroad C, Bart A, Sullivan T, Green C, Fuglie K, Kim G, Surface J, Jermolowicz A, Thomason T *et al*: Aquaculture is agriculture colloquium: USDA's role in supporting farmers of fish, shellfish, and aquatic plants. In*.*: United States Department of Agriculture (USDA); 2021.

[2] Abdelrahman H, ElHady M, Alcivar-Warren A, Allen S, Al-Tobasei R, Bao L, Beck B, Blackburn H, Bosworth B, Buchanan J, et al. Aquaculture genomics, genetics and breeding in the United States: current status, challenges, and priorities for future research. BMC Genomics. 2017;18:1–23. 10.1186/s12864-017-3557-1. 28219347 10.1186/s12864-017-3557-1 PMC5319170

[3] Rexroad C, Vallet J, Matukumalli LK, Reecy J, Bickhart D, Blackburn H, Boggess M, Cheng H, Clutter A, Cockett N. Genome to phenome: improving animal health, production, and well-being–a new USDA blueprint for animal genome research 2018–2027. Front Genet. 2019;10:327. 10.3389/fgene.2019.00327. 31156693 10.3389/fgene.2019.00327 PMC6532451

[4] Houston RD, Bean TP, Macqueen DJ, Gundappa MK, Jin YH, Jenkins TL, Selly SLC, Martin SAM, Stevens JR, Santos EM, et al. Harnessing genomics to fast-track genetic improvement in aquaculture. Nat Rev Genet. 2020;21(7):389–409. 10.1038/s41576-020-0227-y. 32300217 10.1038/s41576-020-0227-y

[5] Huang Y, Li Z, Li M, Zhang X, Shi Q, Xu Z. Fish genomics and its application in disease-resistance breeding. Rev Aquac. 2024. 10.1111/raq.12973.

[6] Yáñez JM, Barría A, López ME, Moen T, Garcia BF, Yoshida GM, Xu P. Genome-wide association and genomic selection in aquaculture. Rev Aquac. 2022;15(2):645–75. 10.1111/raq.12750.

[7] Wang J, Cheng Y, Su B, Dunham RA. Genome manipulation advances in selected aquaculture organisms. Rev Aquac. 2024. 10.1111/raq.12988.

[8] Hollenbeck CM, Johnston IA. Genomic tools and selective breeding in molluscs. Front Genet. 2018;9:253. 10.3389/fgene.2018.00253. 30073016 10.3389/fgene.2018.00253 PMC6058216

[9] Zenger KR, Khatkar MS, Jones DB, Khalilisamani N, Jerry DR, Raadsma HW. Genomic selection in aquaculture: Application, limitations and opportunities with special reference to marine shrimp and pearl oysters. Front Genet. 2018;9:693. 10.3389/fgene.2018.00693. 30728827 10.3389/fgene.2018.00693 PMC6351666

[10] Barría A, Benzie JAH, Houston RD, De Koning D-J, de Verdal H. Genomic selection and genome-wide association study for feed-efficiency traits in a farmed Nile Tilapia (*Oreochromis niloticus*) population. Front Genet. 2021;12:737906. 10.3389/fgene.2021.737906. 34616434 10.3389/fgene.2021.737906 PMC8488396

[11] Verbyla KL, Kube PD, Evans BS. Commercial implementation of genomic selection in Tasmanian Atlantic salmon: Scheme evolution and validation. Evol Appl. 2022;15(4):631–44. 10.1111/eva.13304. 35505884 10.1111/eva.13304 PMC9046822

[12] Tsai H-Y, Hamilton A, Tinch AE, Guy DR, Gharbi K, Stear MJ, Matika O, Bishop SC, Houston RD. Genome wide association and genomic prediction for growth traits in juvenile farmed Atlantic salmon using a high density SNP array. BMC Genomics. 2015;16(1):969. 10.1186/s12864-015-2117-9. 26582102 10.1186/s12864-015-2117-9 PMC4652364

[13] Robledo D, Matika O, Hamilton A, Houston RD. Genome-wide association and genomic selection for resistance to amoebic gill disease in Atlantic salmon. G3 (Bethesda). 2018;8(4):1195–203. 10.1534/g3.118.200075. 29420190 10.1534/g3.118.200075 PMC5873910

[14] Vallejo RL, Leeds TD, Gao G, Parsons JE, Martin KE, Evenhuis JP, Fragomeni BO, Wiens GD, Palti Y. Genomic selection models double the accuracy of predicted breeding values for bacterial cold water disease resistance compared to a traditional pedigree-based model in rainbow trout aquaculture. Genet Sel Evol. 2017;49(1):17. 10.1186/s12711-017-0293-6. 28148220 10.1186/s12711-017-0293-6 PMC5289005

[15] Fraslin C, Koskinen H, Nousianen A, Houston RD, Kause A. Genome-wide association and genomic prediction of resistance to *Flavobacterium columnare* in a farmed rainbow trout population. Aquaculture. 2022;557(738332):738332. 10.1016/j.aquaculture.2022.738332.

[16] Yoshida GM, Bangera R, Carvalheiro R, Correa K, Figueroa R, Lhorente JP, Yáñez JM. Genomic prediction accuracy for resistance against Piscirickettsia salmonis in farmed rainbow trout. G3 (Bethesda). 2018;8(2):719–26. 10.1534/g3.117.300499. 29255117 10.1534/g3.117.300499 PMC5919750

[17] Wang H-P, Shen Z-G, Yao H, O'Bryant P, Rapp D. Sex determination and monosex female production in yellow perch. In: Sex Control in Aquaculture. Chichester: John Wiley & Sons, Ltd; 2018. p. 429–443. 10.1002/9781119127291.ch20.

[18] Xu L, Zhao M, Ryu JH, Hayman ES, Fairgrieve WT, Zohar Y, Luckenbach JA, Wong T-T. Reproductive sterility in aquaculture: A review of induction methods and an emerging approach with application to Pacific Northwest finfish species. Rev Aquac. 2023;15(1):220–41. 10.1111/raq.12712.

[19] Nam S-E, Bae D-Y, Ki J-S, Ahn C-Y, Rhee J-S. The importance of multi-omics approaches for the health assessment of freshwater ecosystems. Mol Cell Toxicol. 2023;19(1):3–11. 10.1007/s13273-022-00286-2.

[20] Cusa M. St John Glew K, Trueman C, Mariani S, Buckley L, Neat F, Longo C: A future for seafood point-of-origin testing using DNA and stable isotope signatures. Rev Fish Biol Fish. 2022;32(2):597–621. 10.1007/s11160-021-09680-w.

[21] Wei S, Yun B, Liu S, Ding T. Multiomics technology approaches in blue foods. Curr Opin Food Sci. 2022;45(100833):100833. 10.1016/j.cofs.2022.100833.

[22] Valenza-Troubat N, Hilario E, Montanari S, Morrison-Whittle P, Ashton D, Ritchie P, Wellenreuther M. Evaluating new species for aquaculture: A genomic dissection of growth in the New Zealand silver trevally (*Pseudocaranx georgianus*). Evol Appl. 2022;15(4):591–602. 10.1111/eva.13281. 35505891 10.1111/eva.13281 PMC9046765

[23] Shivam S, El-Matbouli M, Kumar G. Development of fish parasite vaccines in the OMICs era: Progress and opportunities. Vaccines (Basel). 2021;9(2):179. 10.3390/vaccines9020179. 33672552 10.3390/vaccines9020179 PMC7923790

[24] Bledsoe JW, Small BC. Finfish Microbiota and direct-fed microbial applications in aquaculture. In: Direct-Fed Microbials and Prebiotics for Animals. Cham: Springer International Publishing; 2023. p. 249–300. 10.1007/978-3-031-40512-9_10.

[25] Older CE, Griffin MJ, Richardson BM, Waldbieser GC, Reifers JG, Goodman PM, Ware C, Gatlin DM 3rd, Wise DJ, Yamamoto FY. Influence of probiotic and prebiotic supplementation on intestinal microbiota and resistance to *Edwardsiella ictaluri *infection in channel catfish (*Ictalurus punctatus*) following florfenicol administration. J Fish Dis. 2024;47(4):e13910. 10.1111/jfd.13910. 38153008 10.1111/jfd.13910

[26] Older CE, Richardson BM, Wood M, Waldbieser GC, Ware C, Griffin MJ, Ott BD. Evaluating nanopore sequencing for microbial community characterization in catfish pond water. J World Aquaculture Soc. 2024;55(1):289–301. 10.1111/jwas.13002.

[27] Older CE, Yamamoto FY, Griffin MJ, Ware C, Heckman TI, Soto E, Bosworth BG, Waldbieser GC. Comparison of high-throughput sequencing methods for bacterial microbiota profiling in catfish aquaculture. N Am J Aquac. 2023. 10.1002/naaq.10309.

[28] Cai J, Chan HL, Yan X, Leung P. A global assessment of species diversification in aquaculture. Aquaculture. 2023;576(739837):739837. 10.1016/j.aquaculture.2023.739837.

[29] Bashura J, Burke M, Lapitan R, Mikulec J, Owens T, Reading BJ, Richt JA, Spencer D, Valdivia-Granda W, Weekes J et al. Treats to food and agricultural resources. In*.* Edited by United States DoHS, Office of Intelligence and Analysis (I&A) Public-Private Analytic Exchange Program (AEP); 2021.

[30] Miga KH, Eichler EE. Envisioning a new era: complete genetic information from routine, telomere-to-telomere genomes. Am J Human Genet. 2023;110(11):1832–40. 10.1016/j.ajhg.2023.09.011. 37922882 10.1016/j.ajhg.2023.09.011 PMC10645551

[31] Flores A-M, Christensen KA, Campbell B, Koop BF, Taylor JS. Sablefish (*Anoplopoma fimbria*) chromosome-level genome assembly. G3: Genes, Genomes, Genetics 2023;13(7). 10.1093/g3journal/jkad089. 10.1093/g3journal/jkad089 PMC10320756 37097026

[32] Penaloza C, Gutierrez AP, Eöry L, Wang S, Guo X, Archibald AL, Bean TP, Houston RD. A chromosome-level genome assembly for the Pacific oyster *Crassostrea gigas*. GigaScience. 2021;10(3):giab020. 10.1093/gigascience/giab020. 10.1093/gigascience/giab020 PMC7992393 33764468

[33] Mrowicki R, Uhl R. The genome sequence of the Pacific oyster, *Magallana gigas* (Thunberg, 1793). Wellcome Open Res. 2024;9:284. 10.12688/wellcomeopenres.22255.1. 10.12688/wellcomeopenres.22255.2 PMC11267148 39050697

[34] Puritz JB, Guo X, Hare M, He Y, Hillier LW, Jin S, Liu M, Lotterhos KE, Minx P, Modak T. A second unveiling: Haplotig masking of the eastern oyster genome improves population-level inference. Mol Ecol Resour. 2024;24(1):e13801. 10.1111/1755-0998.13801. 37186213 10.1111/1755-0998.13801

[35] Gao G, Waldbieser GC, Youngblood RC, Zhao D, Pietrak MR, Allen MS, Stannard JA, Buchanan JT, Long RL, Milligan M. The generation of the first chromosome-level de novo genome assembly and the development and validation of a 50K SNP array for the St. John River aquaculture strain of North American Atlantic salmon. G3: Genes, Genomes, Genetics. 2023;13(9):jkad138. 10.1093/g3journal/jkad138. 10.1093/g3journal/jkad138 PMC10468304 37335943

[36] Etherington GJ, Nash W, Ciezarek A, Mehta TK, Barria A, Peñaloza C, Khan MGQ, Durrant A, Forrester N, Fraser F et al. Chromosome-level genome sequence of the Genetically Improved Farmed Tilapia (GIFT, *Oreochromis niloticus*) highlights regions of introgression with O. mossambicus. BMC Genomics. 2022;23(1):832. 10.1186/s12864-022-09065-8. 10.1186/s12864-022-09065-8 PMC9756657 36522771

[37] Peng M, Chen X, Yang C, Liu Q, Li Q, Zhang B, Wang H, Zhu W, Feng P, Zeng D, et al. A high-quality genome assembly of the Pacific white shrimp (*Litopenaeus vannamei*) provides insights into its evolution and adaptation. Aquac Rep. 2023;33(101859):101859. 10.1016/j.aqrep.2023.101859.

[38] Gao G, Magadan S, Waldbieser GC, Youngblood RC, Wheeler PA, Scheffler BE, Thorgaard GH, Palti Y. A long reads-based *de-novo* assembly of the genome of the Arlee homozygous line reveals chromosomal rearrangements in rainbow trout. G3 Genes|Genomes|Genetics. 2021;11(4). 10.1093/g3journal/jkab052. 10.1093/g3journal/jkab052 PMC8763230 33616628

[39] Liu Z, Liu S, Yao J, Bao L, Zhang J, Li Y, Jiang C, Sun L, Wang R, Zhang Y, et al. The channel catfish genome sequence provides insights into the evolution of scale formation in teleosts. Nat Comm. 2016;7(1). 10.1038/ncomms11757. 10.1038/ncomms11757 PMC4895719 27249958

[40] Waldbieser GC, Liu S, Yuan Z, Older CE, Gao D, Shi C, Bosworth BG, Li N, Bao L, Kirby MA, et al. Reference genomes of channel catfish and blue catfish reveal multiple pericentric chromosome inversions. BMC Biol. 2023;21(1). 10.1186/s12915-023-01556-8. 10.1186/s12915-023-01556-8 PMC10071708 37013528

[41] Wang H, Su B, Butts IAE, Dunham RA, Wang X. Chromosome-level assembly and annotation of the blue catfish *Ictalurus furcatus*, an aquaculture species for hybrid catfish reproduction, epigenetics, and heterosis studies. GigaScience. 2022;11. 10.1093/gigascience/giac070. 10.1093/gigascience/giac070 PMC9270728 35809049

[42] Pettersson ME, Rochus CM, Han F, Chen J, Hill J, Wallerman O, Fan G, Hong X, Xu Q, Zhang H, et al. A chromosome-level assembly of the Atlantic herring genome—detection of a supergene and other signals of selection. Genome Res. 2019;29(11):1919–28. 10.1101/gr.253435.119. 31649060 10.1101/gr.253435.119 PMC6836730

[43] Mérot C, Stenløkk KSR, Venney C, Laporte M, Moser M, Normandeau E, Árnyasi M, Kent M, Rougeux C, Flynn JM, et al. Genome assembly, structural variants, and genetic differentiation between lake whitefish young species pairs (*Coregonus *sp.) with long and short reads. Mol Ecol. 2022;32(6):1458–77. 10.1111/mec.16468. 10.1111/mec.16468 35416336

[44] Gómez-Chiarri M, Warren WC, Guo X, Proestou D. Developing tools for the study of molluscan immunity: The sequencing of the genome of the eastern oyster. *Crassostrea virginica.* Fish & Shellfish Immunology. 2015;46(1):2–4. 10.1016/j.fsi.2015.05.004. 25982405 10.1016/j.fsi.2015.05.004

[45] Rondeau EB, Minkley DR, Leong JS, Messmer AM, Jantzen JR, von Schalburg KR, Lemon C, Bird NH, Koop BF. The Genome and Linkage Map of the Northern Pike (*Esox lucius*): Conserved Synteny Revealed between the Salmonid Sister Group and the Neoteleostei. PLoS ONE. 2014;9(7):e102089. 10.1371/journal.pone.0102089. 25069045 10.1371/journal.pone.0102089 PMC4113312

[46] Rhie A, McCarthy SA, Fedrigo O, Damas J, Formenti G, Koren S, Uliano-Silva M, Chow W, Fungtammasan A, Kim J, et al. Towards complete and error-free genome assemblies of all vertebrate species. Nature. 2021;592(7856):737–46. 10.1038/s41586-021-03451-0. 33911273 10.1038/s41586-021-03451-0 PMC8081667

[47] Reid BN, Moran RL, Kopack CJ, Fitzpatrick SW. Rapture-ready darters: Choice of reference genome and genotyping method (whole-genome or sequence capture) influence population genomic inference in *Etheostoma*. Mol Ecol Resour. 2020;21(2):404–20. 10.1111/1755-0998.13275. 33058399 10.1111/1755-0998.13275

[48] Tørresen OK, Star B, Jentoft S, Reinar WB, Grove H, Miller JR, Walenz BP, Knight J, Ekholm JM, Peluso P, et al. An improved genome assembly uncovers prolific tandem repeats in Atlantic cod. BMC Genomics. 2017;18(1). 10.1186/s12864-016-3448-x. 10.1186/s12864-016-3448-x PMC5241972 28100185

[49] Griffiths JS, Sahasrabudhe RM, Marimuthu MPA, Chumchim N, Nguyen OH, Beraut E, Escalona M, Whitehead A. A draft reference genome of the red abalone, *Haliotis rufescens*, for conservation genomics. J Hered. 2022;113(6):673–80. 10.1093/jhered/esac047. 36190478 10.1093/jhered/esac047 PMC9709998

[50] Einfeldt AL, Kess T, Messmer A, Duffy S, Wringe BF, Fisher J, den Heyer C, Bradbury IR, Ruzzante DE, Bentzen P. Chromosome level reference of Atlantic halibut *Hippoglossus hippoglossus* provides insight into the evolution of sexual determination systems. Mol Ecol Resour. 2021;21(5):1686–96. 10.1111/1755-0998.13369. 33655659 10.1111/1755-0998.13369

[51] Jasonowicz AJ, Simeon A, Zahm M, Cabau C, Klopp C, Roques C, Iampietro C, Lluch J, Donnadieu C, Parrinello H, et al. Generation of a chromosome-level genome assembly for Pacific halibut (*Hippoglossus stenolepis*) and characterization of its sex-determining genomic region. Mol Ecol Resour. 2022;22(7):2685–700. 10.1111/1755-0998.13641. 35569134 10.1111/1755-0998.13641 PMC9541706

[52] Polinski JM, Zimin AV, Clark KF, Kohn AB, Sadowski N, Timp W, Ptitsyn A, Khanna P, Romanova DY, Williams P, et al. The American lobster genome reveals insights on longevity, neural, and immune adaptations. Sci Adv. 2021;7(26). 10.1126/sciadv.abe8290. 10.1126/sciadv.abe8290 PMC8221624 34162536

[53] Braasch I, Gehrke AR, Smith JJ, Kawasaki K, Manousaki T, Pasquier J, Amores A, Desvignes T, Batzel P, Catchen J, et al. The spotted gar genome illuminates vertebrate evolution and facilitates human-teleost comparisons. Nat Genet. 2016;48(4):427–37. 10.1038/ng.3526. 26950095 10.1038/ng.3526 PMC4817229

[54] Song H, Guo X, Sun L, Wang Q, Han F, Wang H, Wray GA, Davidson P, Wang Q, Hu Z, et al. The hard clam genome reveals massive expansion and diversification of inhibitors of apoptosis in Bivalvia. BMC Biol. 2021;19(1). 10.1186/s12915-020-00943-9. 10.1186/s12915-020-00943-9 PMC7831173 33487168

[55] Farhat S, Bonnivard E, Pales Espinosa E, Tanguy A, Boutet I, Guiglielmoni N, Flot J-F, Allam B. Comparative analysis of the *Mercenaria mercenaria* genome provides insights into the diversity of transposable elements and immune molecules in bivalve mollusks. BMC Genomics. 2022;23(1). 10.1186/s12864-021-08262-1. 10.1186/s12864-021-08262-1 PMC8905726 35260071

[56] Sun C, Li J, Dong J, Niu Y, Hu J, Lian J, Li W, Li J, Tian Y, Shi Q, et al. Chromosome-level genome assembly for the largemouth bass *Micropterus salmoides* provides insights into adaptation to fresh and brackish water. Mol Ecol Resour. 2020;21(1):301–15. 10.1111/1755-0998.13256. 32985096 10.1111/1755-0998.13256

[57] Sun B, Huang Y, Castro LFC, Yang S, Huang S, Jin W, Zhou H, Ijiri S, Luo Y, Gao J, et al. The chromosome-level genome and key genes associated with mud-dwelling behavior and adaptations of hypoxia and noxious environments in loach (*Misgurnus anguillicaudatus*). BMC Biol. 2023;21(1). 10.1186/s12915-023-01517-1. 10.1186/s12915-023-01517-1 PMC9893644 36726103

[58] Shekhar MS, Katneni VK, Jangam AK, Krishnan K, Prabhudas SK, Jani Angel JR, Sukumaran K, Kailasam M, Jena J. First Report of Chromosome-Level Genome Assembly for Flathead Grey Mullet, *Mugil cephalus* (Linnaeus, 1758). Front Genet. 2022;13. 10.3389/fgene.2022.911446. 10.3389/fgene.2022.911446 PMC9247318 35783261

[59] Christensen KA, Rondeau EB, Sakhrani D, Biagi CA, Johnson H, Joshi J, Flores A-M, Leelakumari S, Moore R, Pandoh PK, et al. The pink salmon genome: Uncovering the genomic consequences of a two-year life cycle. PLoS ONE. 2021;16(12):e0255752. 10.1371/journal.pone.0255752. 34919547 10.1371/journal.pone.0255752 PMC8682878

[60] Rondeau EB, Christensen KA, Sakhrani D, Biagi CA, Wetklo M, Johnson HA, Despins CA, Leggatt RA, Minkley DR, Withler RE, et al. Genome assembly, transcriptome and SNP database for chum salmon (*Oncorhynchus keta*). In.: Cold Spring Harbor Laboratory; 2021. 10.1101/2021.12.27.474290.

[61] Rondeau EB, Christensen KA, Johnson HA, Sakhrani D, Biagi CA, Wetklo M, Despins CA, Leggatt RA, Minkley DR, Withler RE, et al. Insights from a chum salmon (*Oncorhynchus keta*) genome assembly regarding whole-genome duplication and nucleotide variation influencing gene function. G3 (Bethesda). 2023;13(8). 10.1093/g3journal/jkad127. 10.1093/g3journal/jkad127 PMC10411575 37293843

[62] Kim J-H, Leong JS, Koop BF, Devlin RH. Multi-tissue transcriptome profiles for coho salmon (*Oncorhynchus kisutch*), a species undergoing rediploidization following whole-genome duplication. Mar Genomics. 2016;25:33–7. 10.1016/j.margen.2015.11.008. 26614614 10.1016/j.margen.2015.11.008

[63] Christensen KA, Rondeau EB, Minkley DR, Sakhrani D, Biagi CA, Flores A-M, Withler RE, Pavey SA, Beacham TD, Godin T, et al. The sockeye salmon genome, transcriptome, and analyses identifying population defining regions of the genome. PLoS ONE. 2020;15(10):e0240935. 10.1371/journal.pone.0240935. 33119641 10.1371/journal.pone.0240935 PMC7595290

[64] Christensen KA, Leong JS, Sakhrani D, Biagi CA, Minkley DR, Withler RE, Rondeau EB, Koop BF, Devlin RH. Chinook salmon (*Oncorhynchus tshawytscha*) genome and transcriptome. PLoS ONE. 2018;13(4):e0195461. 10.1371/journal.pone.0195461. 29621340 10.1371/journal.pone.0195461 PMC5886536

[65] Conte MA, Joshi R, Moore EC, Nandamuri SP, Gammerdinger WJ, Roberts RB, Carleton KL, Lien S, Kocher TD. Chromosome-scale assemblies reveal the structural evolution of African cichlid genomes. GigaScience. 2019;8(4). 10.1093/gigascience/giz030. 10.1093/gigascience/giz030 PMC6447674 30942871

[66] Gundappa MK, Peñaloza C, Regan T, Boutet I, Tanguy A, Houston RD, Bean TP, Macqueen DJ. Chromosome‐level reference genome for European flat oyster (*Ostrea edulis* L.). Evol Appl. 2022;15(11):1713–29. 10.1111/eva.13460. 10.1111/eva.13460 PMC9679249 36426132

[67] Van Quyen D, Gan HM, Lee YP, Nguyen DD, Nguyen TH, Tran XT, Nguyen VS, Khang DD, Austin CM. Improved genomic resources for the black tiger prawn (*Penaeus monodon*). Mar Genomics. 2020;52:100751. 10.1016/j.margen.2020.100751. 32033920 10.1016/j.margen.2020.100751

[68] Zhang X, Yuan J, Sun Y, Li S, Gao Y, Yu Y, Liu C, Wang Q, Lv X, Zhang X et al. Penaeid shrimp genome provides insights into benthic adaptation and frequent molting. Nat Comm. 2019;10(1). 10.1038/s41467-018-08197-4. 10.1038/s41467-018-08197-4 PMC6341167 30664654

[69] Feron R, Zahm M, Cabau C, Klopp C, Roques C, Bouchez O, Eché C, Valière S, Donnadieu C, Haffray P, et al. Characterization of a Y-specific duplication/insertion of the anti-Mullerian hormone type II receptor gene based on a chromosome-scale genome assembly of yellow perch *Perca flavescens*. Mol Ecol Resour. 2020;20(2):531–43. 10.1111/1755-0998.13133. 10.1111/1755-0998.13133 PMC7050324 31903688

[70] Xu Z, Gao T, Xu Y, Li X, Li J, Lin H, Yan W, Pan J, Tang J. A chromosome-level reference genome of red swamp crayfish *Procambarus clarkii* provides insights into the gene families regarding growth or development in crustaceans. Genomics. 2021;113(5):3274–84. 10.1016/j.ygeno.2021.07.017. 34303807 10.1016/j.ygeno.2021.07.017

[71] Xu R, Martelossi J, Smits M, Iannello M, Peruzza L, Babbucci M, Milan M, Dunham JP, Breton S, Milani L, et al. Multi-tissue RNA-Seq Analysis and Long-read-based Genome Assembly Reveal Complex Sex-specific Gene Regulation and Molecular Evolution in the Manila Clam. Genome Biol Evol. 2022;14(12). 10.1093/gbe/evac171. 10.1093/gbe/evac171 PMC9803972 36508337

[72] Lien S, Koop BF, Sandve SR, Miller JR, Kent MP, Nome T, Hvidsten TR, Leong JS, Minkley DR, Zimin A, et al. The Atlantic salmon genome provides insights into rediploidization. Nature. 2016;533(7602):200–5. 10.1038/nature17164. 27088604 10.1038/nature17164 PMC8127823

[73] Pasquier J, Cabau C, Nguyen T, Jouanno E, Severac D, Braasch I, Journot L, Pontarotti P, Klopp C, Postlethwait JH, et al. Gene evolution and gene expression after whole genome duplication in fish: the PhyloFish database. BMC Genomics. 2016;17(1). 10.1186/s12864-016-2709-z. 10.1186/s12864-016-2709-z PMC4870732 27189481

[74] Araki K. Aokic J-y, Kawase J, Hamada K, Ozaki A, Fujimoto H, Yamamoto I, Usuki H: Whole Genome Sequencing of Greater Amberjack (*Seriola dumerili*) for SNP Identification on Aligned Scaffolds and Genome Structural Variation Analysis Using Parallel Resequencing. International Journal of Genomics. 2018;2018:1–12. 10.1155/2018/7984292. 10.1155/2018/7984292 PMC5896239 29785397

[75] Purcell CM, Seetharam AS, Snodgrass O, Ortega-García S, Hyde JR, Severin AJ. Insights into teleost sex determination from the *Seriola dorsalis* genome assembly. BMC Genomics. 2018;19(1). 10.1186/s12864-017-4403-1. 10.1186/s12864-017-4403-1 PMC5759298 29310588

[76] Ramesh B, Small CM, Healey H, Johnson B, Barker E, Currey M, Bassham S, Myers M, Cresko WA, Jones AG. Improvements to the Gulf pipefish *Syngnathus scovelli* genome. Gigabyte. 2023;2023:1–11. 10.46471/gigabyte.76. 10.46471/gigabyte.76 PMC10038202 36969711

[77] Ciezarek AG, Dunning LT, Jones CS, Noble LR, Humble E, Stefanni SS, Savolainen V. Substitutions in the Glycogenin-1 Gene Are Associated with the Evolution of Endothermy in Sharks and Tunas. Genome Biol Evol. 2016;8(9):3011–21. 10.1093/gbe/evw211. 27614233 10.1093/gbe/evw211 PMC5630876

[78] Wu B, Feng C, Zhu C, Xu W, Yuan Y, Hu M, Yuan K, Li Y, Ren Y, Zhou Y, et al. The Genomes of Two Billfishes Provide Insights into the Evolution of Endothermy in Teleosts. Mol Biol Evol. 2021;38(6):2413–27. 10.1093/molbev/msab035. 33533895 10.1093/molbev/msab035 PMC8136490

[79] Liu S, Martin KE, Snelling WM, Long R, Leeds TD, Vallejo RL, Wiens GD, Palti Y. Accurate genotype imputation from low-coverage whole-genome sequencing data of rainbow trout. G3: Genes, Genomes, Genetics 2024;14(9):jkae168. 10.1093/g3journal/jkae168. 10.1093/g3journal/jkae168 PMC11373650 39041837

[80] Pearse DE, Barson NJ, Nome T, Gao G, Campbell MA, Abadía-Cardoso A, Anderson EC, Rundio DE, Williams TH, Naish KA, et al. Sex-dependent dominance maintains migration supergene in rainbow trout. Nat Ecol Evol. 2019;3(12):1731–42. 10.1038/s41559-019-1044-6. 31768021 10.1038/s41559-019-1044-6

[81] Liu S, Gao G, Layer RM, Thorgaard GH, Wiens GD, Leeds TD, Martin KE, Palti Y. Identification of high-confidence structural variants in domesticated rainbow trout using whole-genome sequencing. Front Genet. 2021;12. 10.3389/fgene.2021.639355. 10.3389/fgene.2021.639355 PMC7959816 33732289

[82] Modak TH, Literman R, Puritz JB, Johnson KM, Roberts EM, Proestou D, Guo X, Gomez-Chiarri M, Schwartz RS. Extensive genome-wide duplications in the eastern oyster (*Crassostrea virginica*). Philos Trans R Soc Lond B Biol Sci. 1825;2021(376):20200164. 10.1098/rstb.2020.0164. 10.1098/rstb.2020.0164 PMC8059967 33813893

[83] Gutierrez AP, Bean TP, Hooper C, Stenton CA, Sanders MB, Paley RK, Rastas P, Bryrom M, Matika O, Houston RD. A genome-wide association study for host resistance to ostreid herpesvirus in Pacific oysters (*Crassostrea gigas*). G3: Genes, Genomes, Genetics. 2018;8(4):1273–80. 10.1534/g3.118.200113. 10.1534/g3.118.200113 PMC5873916 29472307

[84] McCarty AJ, Allen Jr SK, Plough LV. Genome-wide analysis of acute low salinity tolerance in the eastern oyster *Crassostrea virginica* and potential of genomic selection for trait improvement. G3. 2022;12(1):jkab368. 10.1093/g3journal/jkab368. 10.1093/g3journal/jkab368 PMC8727987 34849774

[85] Li N, Zhou T, Geng X, Jin Y, Wang X, Liu S, Xu X, Gao D, Li Q, Liu Z. Identification of novel genes significantly affecting growth in catfish through GWAS analysis. Mol Genet Genomics. 2018;293:587–99. 10.1007/s00438-017-1406-1. 29230585 10.1007/s00438-017-1406-1

[86] Reis Neto RV, Yoshida GM, Lhorente JP, Yáñez JM. Genome-wide association analysis for body weight identifies candidate genes related to development and metabolism in rainbow trout (*Oncorhynchus mykiss*). Mol Genet Genomics. 2019;294:563–71. 10.1007/s00438-018-1518-2. 30635785 10.1007/s00438-018-1518-2

[87] Sánchez-Roncancio C, García B, Gallardo-Hidalgo J, Yáñez JM. GWAS on imputed whole-genome sequence variants reveal genes associated with resistance to *Piscirickettsia salmonis* in rainbow trout (*Oncorhynchus mykiss*). Genes. 2022;14(1):114. 10.3390/genes14010114. 36672855 10.3390/genes14010114 PMC9859203

[88] Tan S, Zhou T, Wang W, Jin Y, Wang X, Geng X, Luo J, Yuan Z, Yang Y, Shi H. GWAS analysis using interspecific backcross progenies reveals superior blue catfish alleles responsible for strong resistance against enteric septicemia of catfish. Mol Genet Genomics. 2018;293:1107–20. 10.1007/s00438-018-1443-4. 29737402 10.1007/s00438-018-1443-4

[89] Ahmed RO, Ali A, Al-Tobasei R, Leeds T, Kenney B, Salem M. Weighted single-step GWAS identifies genes influencing fillet color in rainbow trout. Genes. 2022;13(8):1331. 10.3390/genes13081331. 35893068 10.3390/genes13081331 PMC9332390

[90] Al-Tobasei R, Ali A, Garcia AL, Lourenco D, Leeds T, Salem M. Genomic predictions for fillet yield and firmness in rainbow trout using reduced-density SNP panels. BMC Genomics. 2021;22:1–11. 10.1186/s12864-021-07404-9. 33516179 10.1186/s12864-021-07404-9 PMC7847601

[91] Salem M, Al-Tobasei R, Ali A, Lourenco D, Gao G, Palti Y, Kenney B, Leeds TD. Genome-wide association analysis with a 50K transcribed gene SNP-chip identifies QTL affecting muscle yield in rainbow trout. Front Genet. 2018;9:387. 10.3389/fgene.2018.00387. 30283492 10.3389/fgene.2018.00387 PMC6157414

[92] Vallejo RL, Liu S, Gao G, Fragomeni BO, Hernandez AG, Leeds TD, Parsons JE, Martin KE, Evenhuis JP, Welch TJ, et al. Similar genetic architecture with shared and unique quantitative trait loci for bacterial cold water disease resistance in two rainbow trout breeding populations. Front Genet. 2017;8. 10.3389/fgene.2017.00156. 10.3389/fgene.2017.00156 PMC5660510 29109734

[93] Vela-Avitúa S, LaFrentz BR, Lozano CA, Shoemaker CA, Ospina-Arango JF, Beck BH, Rye M. Genome-wide association study for *Streptococcus iniae* in Nile tilapia (*Oreochromis niloticus*) identifies a significant QTL for disease resistance. Front Genet. 2023;14:1078381. 10.3389/fgene.2023.1078381. 36936431 10.3389/fgene.2023.1078381 PMC10017449

[94] Su B, Shang M, Grewe PM, Patil JG, Peatman E, Perera DA, Cheng Q, Li C, Weng C-C, Li P. Suppression and restoration of primordial germ cell marker gene expression in channel catfish, *Ictalurus punctatus*, using knockdown constructs regulated by copper transport protein gene promoters: Potential for reversible transgenic sterilization. Theriogenology. 2015;84(9):1499–512. 10.1016/j.theriogenology.2015.07.037. 26341409 10.1016/j.theriogenology.2015.07.037

[95] Elaswad A, Khalil K, Ye Z, Liu Z, Liu S, Peatman E, Odin R, Vo K, Drescher D, Gosh K. Effects of CRISPR/Cas9 dosage on TICAM1 and RBL gene mutation rate, embryonic development, hatchability and fry survival in channel catfish. Sci Rep. 2018;8(1):16499. 10.1038/s41598-018-34738-4. 30405210 10.1038/s41598-018-34738-4 PMC6220201

[96] Khalil K, Elaswad A, Abdelrahman H, Michel M, Chen W, Liu S, Odin R, Ye Z, Drescher D, Vo K. Editing the melanocortin-4 receptor gene in channel catfish using the CRISPR-cas9 system. Fishes. 2023;8(2):116. 10.3390/fishes8020116.

[97] Wargelius A, Leininger S, Skaftnesmo KO, Kleppe L, Andersson E, Taranger GL, Schulz RW, Edvardsen RB. Dnd knockout ablates germ cells and demonstrates germ cell independent sex differentiation in Atlantic salmon. Sci Rep. 2016;6(1):21284. 10.1038/srep21284. 26888627 10.1038/srep21284 PMC4758030

[98] Cleveland BM, Yamaguchi G, Radler LM, Shimizu M. Editing the duplicated insulin-like growth factor binding protein-2b gene in rainbow trout (*Oncorhynchus mykiss*). Sci Rep. 2018;8(1):16054. 10.1038/s41598-018-34326-6. 30375441 10.1038/s41598-018-34326-6 PMC6207780

[99] Coogan M, Alston V, Su B, Khalil K, Elaswad A, Khan M, Simora RM, Johnson A, Xing D, Li S. CRISPR/Cas-9 induced knockout of myostatin gene improves growth and disease resistance in channel catfish (*Ictalurus punctatus*). Aquaculture. 2022;557:738290. 10.1016/j.aquaculture.2022.738290.

[100] Datsomor AK, Olsen RE, Zic N, Madaro A, Bones AM, Edvardsen RB, Wargelius A, Winge P. CRISPR/Cas9-mediated editing of Δ5 and Δ6 desaturases impairs Δ8-desaturation and docosahexaenoic acid synthesis in Atlantic salmon (*Salmo salar* L.). Sci Rep. 2019;9(1):16888. 10.1038/s41598-019-53316-w. 10.1038/s41598-019-53316-w PMC6858459 31729437

[101] Jiang D, Chen J, Fan Z, Tan D, Zhao J, Shi H, Liu Z, Tao W, Li M, Wang D. CRISPR/Cas9-induced disruption of wt1a and wt1b reveals their different roles in kidney and gonad development in Nile tilapia. Dev Biol. 2017;428(1):63–73. 10.1016/j.ydbio.2017.05.017. 28527702 10.1016/j.ydbio.2017.05.017

[102] Kishimoto K, Washio Y, Yoshiura Y, Toyoda A, Ueno T, Fukuyama H, Kato K, Kinoshita M. Production of a breed of red sea bream *Pagrus major* with an increase of skeletal muscle mass and reduced body length by genome editing with CRISPR/Cas9. Aquaculture. 2018;495:415–27. 10.1016/j.aquaculture.2018.05.055.

[103] Raudstein M, Straume AH, Kjærner-Semb E, Barvik M, Ellingsen S, Edvardsen RB. Highly efficient in vivo C-to-T base editing in Atlantic salmon (*Salmo salar*)–A step towards aquaculture precision breeding. Aquaculture. 2024;581:740487. 10.1016/j.aquaculture.2023.740487.

[104] Wang C, Xu J, Kocher TD, Li M, Wang D. CRISPR knockouts of *pmela* and *pmelb* engineered a golden tilapia by regulating relative pigment cell abundance. J Hered. 2022;113(4):398–413. 10.1093/jhered/esac018. 35385582 10.1093/jhered/esac018

[105] López-Porras A, Berg RS, Burgerhout E, Hansen ØJ, Györkei Á, Qiao S-W, Johansen F-E. CRISPR-Cas9/Cas12a-based genome editing in Atlantic cod (*Gadus morhua*). Aquaculture. 2024;581:740440. 10.1016/j.aquaculture.2023.740440.

[106] Kim J, Cho JY, Kim J-W, Kim H-C, Noh JK, Kim Y-O, Hwang H-K, Kim W-J, Yeo S-Y, An CM. CRISPR/Cas9-mediated myostatin disruption enhances muscle mass in the olive flounder *Paralichthys olivaceus*. Aquaculture. 2019;512:734336. 10.1016/j.aquaculture.2019.734336.

[107] Ohama M, Washio Y, Kishimoto K, Kinoshita M, Kato K. Growth performance of myostatin knockout red sea bream *Pagrus major* juveniles produced by genome editing with CRISPR/Cas9. Aquaculture. 2020;529: 735672. 10.1016/j.aquaculture.2020.735672.

[108] Khalil K, Elayat M, Khalifa E, Daghash S, Elaswad A, Miller M, Abdelrahman H, Ye Z, Odin R, Drescher D. Generation of myostatin gene-edited channel catfish (*Ictalurus punctatus*) via zygote injection of CRISPR/Cas9 system. Sci Rep. 2017;7(1):7301. 10.1038/s41598-017-07223-7. 28779173 10.1038/s41598-017-07223-7 PMC5544710

[109] Wang J, Su B, Xing D, Bruce TJ, Li S, Bern L, Shang M, Johnson A, Simora RMC, Coogan M. Generation of eco-friendly and disease-resistant channel catfish (*Ictalurus punctatus*) harboring the alligator cathelicidin gene via CRISPR/Cas9 engineering. Engineering. 2024. 10.1016/j.eng.2023.12.005.

[110] Güralp H, Skaftnesmo KO, Kjærner-Semb E, Straume AH, Kleppe L, Schulz RW, Edvardsen RB, Wargelius A. Rescue of germ cells in dnd crispant embryos opens the possibility to produce inherited sterility in Atlantic salmon. Sci Rep. 2020;10(1):18042. 10.1038/s41598-020-74876-2. 33093479 10.1038/s41598-020-74876-2 PMC7581530

[111] Jin YH, Liao B, Migaud H, Davie A. Physiological impact and comparison of mutant screening methods in piwil2 KO founder Nile tilapia produced by CRISPR/Cas9 system. Sci Rep. 2020;10(1):12600. 10.1038/s41598-020-69421-0. 32724054 10.1038/s41598-020-69421-0 PMC7387559

[112] Mokrani A, Liu S. Harnessing CRISPR/Cas9 system to improve economic traits in aquaculture species. Aquaculture. 2024;579:740279. 10.1016/j.aquaculture.2023.740279.

[113] Wray-Cahen D, Bodnar A, Rexroad C, Siewerdt F, Kovich D. Advancing genome editing to improve the sustainability and resiliency of animal agriculture. CABI Agriculture and Bioscience. 2022;3(1):1–17. 10.1186/s43170-022-00091-w.

[114] Copper S. Following the Framework: Intentional Genomic Alterations in Animals. J Food L & Pol’y. 2022;18:117.

[115] Rudenko L, Plunkett LM, Kornum A, Röcklinsberg H, Sørensen DB, Gjerris M. An overview of the regulation of genetically altered animals in research. Biotech Animals Res. 2024:23–72.

[116] Van Eenennaam AL, De Figueiredo SF, Trott JF, Zilberman D. Genetic engineering of livestock: the opportunity cost of regulatory delay. Annual Review of Animal Biosciences. 2021;9(1):453–78. 10.1146/annurev-animal-061220-023052. 33186503 10.1146/annurev-animal-061220-023052

[117] Watson O, Hayta S. Precision breeding in agriculture and food systems in the United Kingdom. Transgenic Res. 2024. 10.1007/s11248-024-00397-7. 39105945 10.1007/s11248-024-00397-7 PMC11655596

[118] Lemay M-A, Malle S. A practical guide to using structural variants for genome-wide association studies. In: Methods in Molecular Biology. Springer US; 2022. p. 161–172. 10.1007/978-1-0716-2237-7_10. 10.1007/978-1-0716-2237-7_10 35641764

[119] Ruigrok M, Xue B, Catanach A, Zhang M, Jesson L, Davy M, Wellenreuther M. The relative power of structural genomic variation versus SNPs in explaining the quantitative trait growth in the marine teleost *Chrysophrys auratus*. Genes. 2022;13(7):1129. 10.3390/genes13071129. 35885912 10.3390/genes13071129 PMC9320665

[120] Bertolotti AC, Layer RM, Gundappa MK, Gallagher MD, Pehlivanoglu E, Nome T, Robledo D, Kent MP, Røsæg LL, Holen MM, et al. The structural variation landscape in 492 Atlantic salmon genomes. Nat Comm. 2020;11(1). 10.1038/s41467-020-18972-x. 10.1038/s41467-020-18972-x PMC7560756 33056985

[121] Lecomte L, Árnyasi M, Ferchaud AL, Kent M, Lien S, Stenløkk K, Sylvestre F, Bernatchez L, Mérot C. Investigating structural variant, indel and single nucleotide polymorphism differentiation between locally adapted Atlantic salmon populations. Evol Appl. 2024;17(3). 10.1111/eva.13653. 10.1111/eva.13653 PMC10940791 38495945

[122] Gerdol M, Moreira R, Cruz F, Gómez-Garrido J, Vlasova A, Rosani U, Venier P, Naranjo-Ortiz MA, Murgarella M, Balseiro P, et al. Massive gene presence/absence variation in the mussel genome as an adaptive strategy: first evidence of a pan-genome in Metazoa. In.: Cold Spring Harbor Laboratory; 2019. 10.1101/781377.

[123] Johnston IA, Kent MP, Boudinot P, Looseley M, Bargelloni L, Faggion S, Merino GA, Ilsley GR, Bobe J, Tsigenopoulos CS. Advancing fish breeding in aquaculture through genome functional annotation. Aquaculture. 2024:740589. 10.1016/j.aquaculture.2024.740589.

[124] Salem M, Al-Tobasei R, Ali A, An L, Wang Y, Bai X, Bi Y. H Z: Functional annotation of regulatory elements in rainbow trout uncovers roles of the epigenome in genetic selection and genome evolution. GigaScience. 2025;13:1–15. 10.1093/gigascience/giae092. 10.1093/gigascience/giae092 PMC11629980 39657104

[125] NOAA. NOAA Aquaculture Strategic Plan (2023–2028). In: National Oceanic and Atmospheric Administration (NOAA); 2022.

[126] Aquaculture NSaTCSo: A National Strategic Plan for Aquaculture Research. In*.*: National Science and Technology Council Subcommittee on Aquaculture; 2022.

[127] Rexroad C, Rust MB, Riche M, Wills P, Davis M. Opportunities for US marine finfish aquaculture. J World Aquaculture Soc. 2021;52(3):501–8. 10.1111/jwas.12791.

[128] Froehlich HE, Gentry RR, Lester SE, Rennick M, Lemoine HR, Tapia-Lewin S, Gardner L. Piecing together the data of the US marine aquaculture puzzle. J Environ Manage. 2022;308:114623. 10.1016/j.jenvman.2022.114623. 35121466 10.1016/j.jenvman.2022.114623

[129] Kumar G, Hegde S, van Senten J, Engle C, Boldt N, Parker M, Quagrainie K, Posadas B, Asche F, Dey M. Economic contribution of US aquaculture farms. J World Aquacul Soc. 2024:e13091. 10.1111/jwas.13091.

[130] Yang Y, Fu Q, Wang X, Liu Y, Zeng Q, Li Y, Gao S, Bao L, Liu S, Gao D. Comparative transcriptome analysis of the swimbladder reveals expression signatures in response to low oxygen stress in channel catfish *Ictalurus punctatus*. Physiol Genomics. 2018;50(8):636–47. 10.1152/physiolgenomics.00125.2017. 10.1152/physiolgenomics.00125.2017 29799804

[131] Ott BD, Hulse-Kemp AM, Duke MV, Griffin MJ, Peterson BC, Scheffler BE, Torrans EL, Allen PJ. Hypothalamic transcriptome response to simulated diel earthen pond hypoxia cycles in channel catfish (*Ictalurus punctatus*). Physiol Genomics. 2024;56(8):519–30. 10.1152/physiolgenomics.00007.2024. 38808773 10.1152/physiolgenomics.00007.2024

[132] Andersen LK, Abernathy JW, Farmer BD, Lange MD, McEntire ME, Rawles SD. Gene expression profiles of white bass (*Morone chrysops*) and hybrid striped bass (*M. chrysops* x *M. saxatilis*) gill tissue following *Flavobacterium covae* infection. Comparative Immunol Rep. 2024:200144. 10.1016/j.cirep.2024.200144.

[133] Wang R, Sun L, Bao L, Zhang J, Jiang Y, Yao J, Song L, Feng J, Liu S, Liu Z. Bulk segregant RNA-seq reveals expression and positional candidate genes and allele-specific expression for disease resistance against enteric septicemia of catfish. BMC Genomics. 2013;14:1–18. 10.1186/1471-2164-14-929. 24373586 10.1186/1471-2164-14-929 PMC3890627

[134] Wang X, Liu S, Yang Y, Fu Q, Abebe A, Liu Z. Identification of NF-κB related genes in channel catfish and their expression profiles in mucosal tissues after columnaris bacterial infection. Dev Comp Immunol. 2017;70:27–38. 10.1016/j.dci.2017.01.003. 28063885 10.1016/j.dci.2017.01.003

[135] Proestou DA, Sullivan ME. Variation in global transcriptomic response to *Perkinsus marinus* infection among eastern oyster families highlights potential mechanisms of disease resistance. Fish Shellfish Immunol. 2020;96:141–51. 10.1016/j.fsi.2019.12.001. 31809834 10.1016/j.fsi.2019.12.001

[136] Proestou DA, Sullivan ME, Lundgren KM, Ben-Horin T, Witkop EM, Hart KM. Understanding *Crassostrea virginica* tolerance of *Perkinsus marinus* through global gene expression analysis. Front Genet. 2023;14:1054558. 10.3389/fgene.2023.1054558. 36741318 10.3389/fgene.2023.1054558 PMC9892467

[137] Sullivan ME, Proestou DA. Survival and transcriptomic responses to different *Perkinsus marinus* exposure methods in an Eastern oyster family. Aquaculture. 2021;542(736831):736831. 10.1016/j.aquaculture.2021.736831.

[138] Tan S, Wang W, Zhong X, Tian C, Niu D, Bao L, Zhou T, Jin Y, Yang Y, Yuan Z. Increased alternative splicing as a host response to *Edwardsiella ictaluri* infection in catfish. Mar Biotechnol. 2018;20:729–38. 10.1007/s10126-018-9844-2. 10.1007/s10126-018-9844-2 30014301

[139] Tan S, Wang W, Tian C, Niu D, Zhou T, Yang Y, Gao D, Liu Z. Post-transcriptional regulation through alternative splicing after infection with *Flavobacterium columnare* in channel catfish (*Ictalurus punctatus*). Fish Shellfish Immunol. 2019;91:188–93. 10.1016/j.fsi.2019.05.008. 31077849 10.1016/j.fsi.2019.05.008

[140] Tan S, Wang W, Tian C, Niu D, Zhou T, Jin Y, Yang Y, Gao D, Dunham R, Liu Z. Heat stress induced alternative splicing in catfish as determined by transcriptome analysis. Comp Biochem Physiol D: Genomics Proteomics. 2019;29:166–72. 10.1016/j.cbd.2018.11.008. 30481682 10.1016/j.cbd.2018.11.008

[141] Tan S, Wang W, Zhou T, Yang Y, Gao D, Dunham R, Liu Z. Polyadenylation sites and their characteristics in the genome of channel catfish (*Ictalurus punctatus*) as revealed by using RNA-Seq data. Comp Biochem Physiol D: Genomics Proteomics. 2019;30:248–55. 10.1016/j.cbd.2019.03.008. 30952021 10.1016/j.cbd.2019.03.008

[142] Ali A, Thorgaard GH, Salem M. PacBio Iso-Seq improves the rainbow trout genome annotation and identifies alternative splicing associated with economically important phenotypes. Front Genet. 2021;12:683408. 10.3389/fgene.2021.683408. 34335690 10.3389/fgene.2021.683408 PMC8321248

[143] Rajab SA, Andersen LK, Kenter LW, Berlinsky DL, Borski RJ, McGinty AS, Ashwell CM, Ferket PR, Daniels HV, Reading BJ. Combinatorial metabolomic and transcriptomic analysis of muscle growth in hybrid striped bass (female white bass *Morone chrysops *x male striped bass *M. saxatilis*). BMC Genomics. 2024;25(1):580. 10.1186/s12864-024-10325-y. 10.1186/s12864-024-10325-y PMC11165755 38858615

[144] Chapman RW, Reading BJ, Sullivan CV. Ovary transcriptome profiling via artificial intelligence reveals a transcriptomic fingerprint predicting egg quality in striped bass, *Morone saxatilis*. PLoS ONE. 2014;9(5):e96818. 10.1371/journal.pone.0096818. 24820964 10.1371/journal.pone.0096818 PMC4018430

[145] Sullivan CV, Chapman RW, Reading BJ, Anderson PE. Transcriptomics of mRNA and egg quality in farmed fish: Some recent developments and future directions. Gen Comp Endocrinol. 2015;221:23–30. 10.1016/j.ygcen.2015.02.012. 25725305 10.1016/j.ygcen.2015.02.012

[146] Reading BJ, Williams VN, Chapman RW, Williams TI, Sullivan CV. Dynamics of the striped bass (*Morone saxatilis*) ovary proteome reveal a complex network of the translasome. J Proteome Res. 2013;12(4):1691–9. 10.1021/pr3010293. 23414552 10.1021/pr3010293

[147] Schilling J, Nepomuceno A, Schaff JE, Muddiman DC, Daniels HV, Reading BJ. Compartment proteomics analysis of white perch (*Morone americana*) ovary using support vector machines. J Proteome Res. 2014;13(3):1515–26. 10.1021/pr401067g. 24494930 10.1021/pr401067g

[148] Palaiokostas C. Predicting for disease resistance in aquaculture species using machine learning models. Aquaculture Reports. 2021;20: 100660. 10.1016/j.aqrep.2021.100660.

[149] Ellisor DL, Bayless AL, Schock TB, Davis WC, Knott BT, Seghers J, Leys H, Emteborg H. Multi-omics characterization of NIST seafood reference materials and alternative matrix preparations. Anal Bioanal Chem. 2024;416(3):773–85. 10.1007/s00216-023-04928-9. 37723254 10.1007/s00216-023-04928-9

[150] Daniels RR, Taylor RS, Robledo D, Macqueen DJ. Single cell genomics as a transformative approach for aquaculture research and innovation. Rev Aquac. 2023;15(4):1618–37. 10.1111/raq.12806. 38505116 10.1111/raq.12806 PMC10946576

[151] Aldersey JE, Lange MD, Beck BH, Abernathy JW. Single-nuclei transcriptome analysis of channel catfish spleen provides insight into the immunome of an aquaculture-relevant species. PLoS ONE. 2024;19(9):e0309397. 10.1371/journal.pone.0309397. 39325796 10.1371/journal.pone.0309397 PMC11426453

[152] Cao M, Xue T, Huo H, Zhang X, Wang NN, Yan X, Li C. Spatial transcriptomes and microbiota reveal immune mechanism that respond to pathogen infection in the posterior intestine of *Sebastes schlegelii*. Open Biol. 2023;13(2):220302. 10.1098/rsob.220302. 36974664 10.1098/rsob.220302 PMC9944294

[153] Sveen LR, Robinson N, Krasnov A, Daniels RR, Vaadal M, Karlsen C, Ytteborg E, Robledo D, Salisbury S, Dagnachew B et al. Transcriptomic landscape of Atlantic salmon (*Salmo salar* L.) skin. G3 (Bethesda). 2023;13(11). 10.1093/g3journal/jkad215. 10.1093/g3journal/jkad215 PMC10627282 37724757

[154] Taylor RS, Ruiz Daniels R, Dobie R, Naseer S, Clark TC, Henderson NC, Boudinot P, Martin SAM, Macqueen DJ. Single cell transcriptomics of Atlantic salmon (*Salmo salar* L.) liver reveals cellular heterogeneity and immunological responses to challenge by *Aeromonas salmonicida*. Front Immunol. 2022;13:984799. 10.3389/fimmu.2022.984799. 10.3389/fimmu.2022.984799 PMC9450062 36091005

[155] Wang H-P, Shen Z-G. The potential role of epigenetics in aquaculture: insights from different taxa to diverse teleosts. 2023:1–43. 10.1002/9781119821946.ch1.

[156] Piferrer F, Wang H-P. Epigenetics in aquaculture: knowledge gaps, challenges, and future prospects. 2023:451–63. 10.1002/9781119821946.ch20.

[157] Gavery MR, Roberts SB. Epigenetic considerations in aquaculture. PeerJ. 2017;5(e4147):e4147. 10.7717/peerj.4147. 29230373 10.7717/peerj.4147 PMC5723431

[158] Liu Z, Zhou T, Gao D. Genetic and epigenetic regulation of growth, reproduction, disease resistance and stress responses in aquaculture. Front Genet. 2022;13:994471. 10.3389/fgene.2022.994471. 36406125 10.3389/fgene.2022.994471 PMC9666392

[159] Yang Y, Zhou T, Liu Y, Tian C, Bao L, Wang W, Zhang Y, Liu S, Shi H, Tan S, et al. Identification of an epigenetically marked locus within the sex determination region of channel catfish. Int J Mol Sci. 2022;23(10):5471. 10.3390/ijms23105471. 35628283 10.3390/ijms23105471 PMC9171582

[160] Wang W, Yang Y, Tan S, Zhou T, Liu Y, Tian C, Bao L, Xing D, Su B, Wang J. Genomic imprinting-like monoallelic paternal expression determines sex of channel catfish. Sci Adv. 2022;8(51):eadc8786. 10.1126/sciadv.adc8786. 10.1126/sciadv.adc8786 PMC9770954 36542716

[161] Gong G, Xiong Y, Xiao S, Li X-Y, Huang P, Liao Q, Han Q, Lin Q, Dan C, Zhou L. Origin and chromatin remodeling of young X/Y sex chromosomes in catfish with sexual plasticity. Nat Sci Rev. 2023;10(2):nwac239. 10.1093/nsr/nwac239. 10.1093/nsr/nwac239 PMC9945428 36846302

[162] Saito T, Whatmore P, Taylor JF, Fernandes JMO, Adam A-C, Tocher DR, Espe M, Skjærven KH. Micronutrient supplementation affects DNA methylation in male gonads with potential intergenerational epigenetic inheritance involving the embryonic development through glutamate receptor-associated genes. BMC Genomics. 2022;23(1):115. 10.1186/s12864-022-08348-4. 35144563 10.1186/s12864-022-08348-4 PMC8832813

[163] Woods LC III, Li Y, Ding Y, Liu J, Reading BJ, Fuller SA, Song J. DNA methylation profiles correlated to striped bass sperm fertility. BMC Genomics. 2018;19:1–10. 10.1186/s12864-018-4548-6. 29636007 10.1186/s12864-018-4548-6 PMC5894188

[164] Salem M, Al-Tobasei R, Ali A, Kenney B. Integrated analyses of DNA methylation and gene expression of rainbow trout muscle under variable ploidy and muscle atrophy conditions. Genes. 2022;13(7):1151. 10.3390/genes13071151. 35885934 10.3390/genes13071151 PMC9319582

[165] Johnson KM, Sirovy KA, Casas SM, La Peyre JF, Kelly MW. Characterizing the Epigenetic and Transcriptomic Responses to *Perkinsus marinus *Infection in the Eastern Oyster *Crassostrea virginica*. Front Mar Sci. 2020;7. 10.3389/fmars.2020.00598.

[166] Spencer LH, Venkataraman YR, Crim R, Ryan S, Horwith MJ, Roberts SB. Carryover effects of temperature and pCO2 across multiple Olympia oyster populations. Ecol Appl. 2020;30(3):e02060. 10.1002/eap.2060. 31863716 10.1002/eap.2060

[167] Gurr SJ, Wanamaker SA, Vadopalas B, Roberts SB, Putnam HM. Repeat exposure to hypercapnic seawater modifies growth and oxidative status in a tolerant burrowing clam. J Exp Biol. 2021;224(13). 10.1242/jeb.233932. 10.1242/jeb.233932 34027545

[168] Putnam HM, Trigg SA, White SJ, Spencer LH, Vadopalas B, Natarajan A, Hetzel J, Jaeger E, Soohoo J, Gallardo-Escárate C, et al. Dynamic DNA methylation contributes to carryover effects and beneficial acclimatization in geoduck clams. 2022. 10.1101/2022.06.24.497506.

[169] Šrut M. Ecotoxicological epigenetics in invertebrates: Emerging tool for the evaluation of present and past pollution burden. Chemosphere. 2021;282(131026):131026. 10.1016/j.chemosphere.2021.131026. 34111635 10.1016/j.chemosphere.2021.131026

[170] Silliman K, Spencer LH, White SJ, Roberts SB. Epigenetic and genetic population structure is coupled in a marine invertebrate. Genome Biol Evol. 2023;15(2). 10.1093/gbe/evad013. 10.1093/gbe/evad013 PMC10468963 36740242

[171] Jobling M. National Research Council (NRC): Nutrient requirements of fish and shrimp. Aquacult Int. 2011;20(3):601–2. 10.1007/s10499-011-9480-6.

[172] Bonder MJ, Kurilshikov A, Tigchelaar EF, Mujagic Z, Imhann F, Vila AV, Deelen P, Vatanen T, Schirmer M, Smeekens SP, et al. The effect of host genetics on the gut microbiome. Nat Genet. 2016;48(11):1407–12. 10.1038/ng.3663. 27694959 10.1038/ng.3663

[173] Awany D, Allali I, Dalvie S, Hemmings S, Mwaikono KS, Thomford NE, Gomez A, Mulder N, Chimusa ER. Host and microbiome genome-wide association studies: current state and challenges. Front Genet. 2019;9. 10.3389/fgene.2018.00637. 10.3389/fgene.2018.00637 PMC6349833 30723493

[174] Chapagain P, Arivett B, Cleveland BM, Walker DM, Salem M. Analysis of the fecal microbiota of fast- and slow-growing rainbow trout (*Oncorhynchus mykiss*). BMC Genomics. 2019;20(1). 10.1186/s12864-019-6175-2. 10.1186/s12864-019-6175-2 PMC6819385 31664902

[175] Chapagain P, Walker D, Leeds T, Cleveland BM, Salem M. Distinct microbial assemblages associated with genetic selection for high- and low- muscle yield in rainbow trout. BMC Genomics. 2020;21(1). 10.1186/s12864-020-07204-7. 10.1186/s12864-020-07204-7 PMC7684950 33228584

[176] Raymo G, Ali A, Ahmed RO, Salem M. Early-life fecal transplantation from high muscle yield rainbow trout to low muscle yield recipients accelerates somatic growth through respiratory and mitochondrial efficiency modulation. Microorganisms. 2024;12(2):261. 10.3390/microorganisms12020261. 38399665 10.3390/microorganisms12020261 PMC10893187

[177] English G, Lawrence MJ, McKindsey CW, Lacoursière-Roussel A, Bergeron H, Gauthier S, Wringe BF, Trudel M. A review of data collection methods used to monitor the associations of wild species with marine aquaculture sites. Rev Aquac. 2024;16(3):1160–85. 10.1111/raq.12890.

[178] King WL, Siboni N, Williams NL, Kahlke T, Nguyen KV, Jenkins C, Dove M, O’Connor W, Seymour JR, Labbate M. Variability in the composition of Pacific oyster microbiomes across oyster families exhibiting different levels of susceptibility to OsHV-1 μvar disease. Front Microbiol. 2019;10:473. 10.3389/fmicb.2019.00473. 30915058 10.3389/fmicb.2019.00473 PMC6421512

[179] Rieder J, Kapopoulou A, Bank C, Adrian-Kalchhauser I. Metagenomics and metabarcoding experimental choices and their impact on microbial community characterization in freshwater recirculating aquaculture systems. Environmental microbiome. 2023;18(1):8. 10.1186/s40793-023-00459-z. 36788626 10.1186/s40793-023-00459-z PMC9930364

[180] Stevick RJ, Post AF, Gómez-Chiarri M. Functional plasticity in oyster gut microbiomes along a eutrophication gradient in an urbanized estuary. Animal microbiome. 2021;3:1–17. 10.1186/s42523-020-00066-0. 33499983 10.1186/s42523-020-00066-0 PMC7934548

[181] Cardona E, Gueguen Y, Magré K, Lorgeoux B, Piquemal D, Pierrat F, Noguier F, Saulnier D. Bacterial community characterization of water and intestine of the shrimp *Litopenaeus stylirostris* in a biofloc system. BMC Microbiol. 2016;16:1–9. 10.1186/s12866-016-0770-z. 27435866 10.1186/s12866-016-0770-z PMC4952143

[182] Drønen K, Roalkvam I, Dahle H, Olsen AB, Nilsen H, Wergeland H. Microbiome dataset from a marine recirculating aquaculture system (RAS) for salmon post-smolt production in Norway. Data Brief. 2022;40: 107767. 10.1016/j.dib.2021.107767. 35005153 10.1016/j.dib.2021.107767 PMC8718742

[183] Rajeev M, Jung I, Lim Y, Kim S, Kang I, Cho J-C. Metagenome sequencing and recovery of 444 metagenome-assembled genomes from the biofloc aquaculture system. Scientific data. 2023;10(1):707. 10.1038/s41597-023-02622-0. 37848477 10.1038/s41597-023-02622-0 PMC10582022

[184] Stilwell JM, Camus AC, Ware C, Walker CM, Stanton JB, Leary JH, Khoo LH, Wise DJ, Waldbieser GC, Griffin MJ. Influence of channel catfish and channel × blue catfish hybrids on myxozoan community composition in catfish aquaculture ponds. N Am J Aquac. 2023;85(3):241–51. 10.1002/naaq.10293.

[185] Pukk L, Kanefsky J, Heathman AL, Weise EM, Nathan LR, Herbst SJ, Sard NM, Scribner KT, Robinson JD. eDNA metabarcoding in lakes to quantify influences of landscape features and human activity on aquatic invasive species prevalence and fish community diversity. Divers Distrib. 2021;27(10):2016–31. 10.1111/ddi.13370.

[186] Timmins-Schiffman E, White SJ, Thompson RE, Vadopalas B, Eudeline B, Nunn BL, Roberts SB. Coupled microbiome analyses highlights relative functional roles of bacteria in a bivalve hatchery. Environ Microbiome. 2021;16(1):7. 10.1186/s40793-021-00376-z. 33902744 10.1186/s40793-021-00376-z PMC8066469

[187] Chakraborty D, Sharma N, Kour S, Sodhi SS, Gupta MK, Lee SJ, Son YO. Applications of omics technology for livestock selection and improvement. Front Genet. 2022;13:774113. 10.3389/fgene.2022.774113. 35719396 10.3389/fgene.2022.774113 PMC9204716

[188] Yang Y, Saand MA, Huang L, Abdelaal WB, Zhang J, Wu Y, Li J, Sirohi MH, Wang F. Applications of multi-omics technologies for crop improvement. Front Plant Sci. 2021;12:563953. 10.3389/fpls.2021.563953. 34539683 10.3389/fpls.2021.563953 PMC8446515

[189] López de Maturana E, Alonso L, Alarcón P, Martín-Antoniano IA, Pineda S, Piorno L, Calle ML, Malats N. Challenges in the integration of omics and non-omics data. Genes. 2019;10(3):238. 10.3390/genes10030238. 10.3390/genes10030238 PMC6471713 30897838

[190] Graw S, Chappell K, Washam CL, Gies A, Bird J, Robeson MS, Byrum SD. Multi-omics data integration considerations and study design for biological systems and disease. Molecular omics. 2021;17(2):170–85. 10.1039/D0MO00041H. 33347526 10.1039/d0mo00041h PMC8058243

[191] Subramanian I, Verma S, Kumar S, Jere A, Anamika K. Multi-omics data integration, interpretation, and its application. Bioinform Biol Insights. 2020;14:1177932219899051. 32076369 10.1177/1177932219899051 PMC7003173

[192] Madrid-Márquez L, Rubio-Escudero C, Pontes B, González-Pérez A, Riquelme JC, Sáez ME. MOMIC: A multi-omics pipeline for data analysis, integration and interpretation. Appl Sci. 2022;12(8):3987. 10.3390/app12083987.

[193] Miao B-B, Dong W, Gu Y-X, Han Z-F, Luo X, Ke C-H, You W-W. OmicsSuite: a customized and pipelined suite for analysis and visualization of multi-omics big data. Horticulture Res. 2023;10(11). 10.1093/hr/uhad195. 10.1093/hr/uhad195 PMC10673651 38023482

[194] Ramos M, Schiffer L, Re A, Azhar R, Basunia A, Rodriguez C, Chan T, Chapman P, Davis SR, Gomez-Cabrero D, et al. Software for the integration of multiomics experiments in Bioconductor. Can Res. 2017;77(21):e39–42. 10.1158/0008-5472.can-17-0344. 10.1158/0008-5472.CAN-17-0344 PMC5679241 29092936

[195] Pfau T, Galhardo M, Lin J, Sauter T. IDARE2—simultaneous visualisation of multiomics data in cytoscape. Metabolites. 2021;11(5):300. 10.3390/metabo11050300. 34066448 10.3390/metabo11050300 PMC8148105

[196] Shannon P, Markiel A, Ozier O, Baliga NS, Wang JT, Ramage D, Amin N, Schwikowski B, Ideker T. Cytoscape: A software environment for integrated models of biomolecular interaction networks. Genome Res. 2003;13(11):2498–504. 10.1101/gr.1239303. 14597658 10.1101/gr.1239303 PMC403769

[197] Chen T, Abadi AJ, Lê Cao K-A, Tyagi S. Multiomics: A user-friendly multi-omics data harmonisation R pipeline. F1000Research. 2023;10:538. 10.12688/f1000research.53453.2.

[198] Andersen LK, Reading BJ. A supervised machine learning workflow for the reduction of highly dimensional biological data. Artificial Intelligence in the Life Sciences. 2024;5:100090. 10.1016/j.ailsci.2023.100090.

[199] Fu G, Yuna Y. Phenotyping and phenomics in aquaculture breeding. Aquaculture and Fisheries. 2022;7(2):140–6. 10.1016/j.aaf.2021.07.001.

[200] Houle D, Govindaraju DR, Omholt S. Phenomics: the next challenge. Nat Rev Genet. 2010;11(12):855–66. 10.1038/nrg2897. 21085204 10.1038/nrg2897

[201] Pérez-Enciso M, Steibel JP. Phenomes: the current frontier in animal breeding. Genet Sel Evol. 2021;53(1). 10.1186/s12711-021-00618-1. 10.1186/s12711-021-00618-1 PMC7934239 33673800

[202] Fuentes R, Letelier J, Tajer B, Valdivia LE, Mullins MC. Fishing forward and reverse: Advances in zebrafish phenomics. Mech Dev. 2018;154:296–308. 10.1016/j.mod.2018.08.007. 30130581 10.1016/j.mod.2018.08.007 PMC6289646

[203] Wang Z, Liu H, Zhang G, Yang X, Wen L, Zhao W. Diseased fish detection in the underwater environment using an improved YOLOV5 network for intensive aquaculture. Fishes. 2023;8(3):169. 10.3390/fishes8030169.

[204] Babu KM, Bentall D, Ashton DT, Puklowski M, Fantham W, Lin HT, Tuckey NPL, Wellenreuther M, Jesson LK. Computer vision in aquaculture: a case study of juvenile fish counting. J R Soc N Z. 2022;53(1):52–68. 10.1080/03036758.2022.2101484. 39439994 10.1080/03036758.2022.2101484 PMC11459735

[205] Deng Y, Tan H, Tong M, Zhou D, Li Y, Zhu M. An automatic recognition method for fish species and length using an underwater stereo vision system. Fishes. 2022;7(6):326. 10.3390/fishes7060326.

[206] Li D, Hao Y, Duan Y. Nonintrusive methods for biomass estimation in aquaculture with emphasis on fish: a review. Rev Aquac. 2020;12(3):1390–411. 10.1111/raq.12388.

[207] Ranjan R, Tsukuda S, Good C. MortCam: An artificial intelligence-aided fish mortality detection and alert system for recirculating aquaculture. Aquacult Eng. 2023;102:102341. 10.1016/j.aquaeng.2023.102341.

[208] Zhao F, Hao J, Zhang H, Yu X, Yan Z, Wu F. Quality recognition method of oyster based on U-net and random forest. J Food Compos Anal. 2024;125:105746. 10.1016/j.jfca.2023.105746.

[209] Mayormente M. Intelligent recirculating aquaculture system of *Oreochromis niloticus*: A feed-conversion-ratio-based machine learning approach. Int J Intell Syst Appl Eng. 2024;12(13s):122–8.

[210] Graham CA, Shamkhalichenar H, Browning VE, Byrd VJ, Liu Y, Gutierrez-Wing MT, Novelo N, Choi J-W, Tiersch TR. A practical evaluation of machine learning for classification of ultrasound images of ovarian development in channel catfish (*Ictalurus punctatus*). Aquaculture. 2022;552: 738039. 10.1016/j.aquaculture.2022.738039. 35296028 10.1016/j.aquaculture.2022.738039 PMC8920069

[211] Chen Y, Yue J, Li Z, Yang J, Wang W. Pacific oyster gonad identification and grayscale calculation based on unapparent object detection. In: Chinese Conference on Pattern Recognition and Computer Vision (PRCV): 2023: Springer; 2023:94–106.

[212] Guévélou E, Allen SK Jr. Use of near infrared reflectance spectroscopy (NIRS) for the rapid compositional analysis of di-, tri-, and tetraploid eastern oysters (*Crassostrea virginica*). Aquaculture. 2016;459:203–9. 10.1016/j.aquaculture.2016.03.022.

[213] Rather MA, Ahmad I, Shah A, Hajam YA, Amin A, Khursheed S, Ahmad I, Rasool S. Exploring opportunities of artificial intelligence in aquaculture to meet increasing food demand. Food Chem: X. 2024:101309. 10.1016/j.fochx.2024.101309. 10.1016/j.fochx.2024.101309 PMC10972841 38550881

[214] Grassi S, Benedetti S, Casiraghi E, Buratti S. E-sensing systems for shelf life evaluation: A review on applications to fresh food of animal origin. Food Packag Shelf Life. 2023;40:101221. 10.1016/j.fpsl.2023.101221.

[215] Grassi S, Benedetti S, Magnani L, Pianezzola A, Buratti S. Seafood freshness: e-nose data for classification purposes. Food Control. 2022;138:108994. 10.1016/j.foodcont.2022.108994.

[216] Difford GF, Boison SA, Khaw HL, Gjerde B. Validating non-invasive growth measurements on individual Atlantic salmon in sea cages using diode frames. Comput Electron Agric. 2020;173:105411. 10.1016/j.compag.2020.105411.

[217] Macaulay G, Warren-Myers F, Barrett LT, Oppedal F, Føre M, Dempster T. Tag use to monitor fish behaviour in aquaculture: a review of benefits, problems and solutions. Rev Aquac. 2021;13(3):1565–82. 10.1111/raq.12534.

[218] O’Donncha F, Stockwell CL, Planellas SR, Micallef G, Palmes P, Webb C, Filgueira R, Grant J. Data driven insight into fish behaviour and their use for precision aquaculture. Frontiers in Animal Science. 2021;2:695054. 10.3389/fanim.2021.695054.

[219] Triant DA, Walsh AT, Hartley GA, Petry B, Stegemiller MR, Nelson BM, McKendrick MM, Fuller EP, Cockett NE, Koltes JE, et al. AgAnimalGenomes: browsers for viewing and manually annotating farm animal genomes. Mamm Genome. 2023;34(3):418–36. 10.1007/s00335-023-10008-1. 37460664 10.1007/s00335-023-10008-1 PMC10382368

[220] Field D, Amaral-Zettler L, Cochrane G, Cole JR, Dawyndt P, Garrity GM, Gilbert J, Glöckner FO, Hirschman L, Karsch-Mizrachi I, et al. The Genomic Standards Consortium. PLoS Biol. 2011;9(6):e1001088. 10.1371/journal.pbio.1001088. 21713030 10.1371/journal.pbio.1001088 PMC3119656

[221] Lawniczak MKN, Durbin R, Flicek P, Lindblad-Toh K, Wei X, Archibald JM, Baker WJ, Belov K, Blaxter ML, Marques Bonet T et al. Standards recommendations for the Earth BioGenome Project. Proc Nat Acad Sci. 2022;119(4).10.1073/pnas.2115639118. 10.1073/pnas.2115639118 PMC8795494 35042802

[222] Abascal F, Acosta R, Addleman NJ, Adrian J, Afzal V, Aken B, Ai R, Akiyama JA, Jammal OA, Amrhein H, et al. Perspectives on ENCODE. Nature. 2020;583(7818):693–8. 10.1038/s41586-020-2449-8. 32728248 10.1038/s41586-020-2449-8 PMC7410827

[223] Consortium EP. A User’s Guide to the Encyclopedia of DNA Elements (ENCODE). PLoS Biol. 2011;9(4):e1001046. 10.1371/journal.pbio.1001046. 21526222 10.1371/journal.pbio.1001046 PMC3079585

[224] Wilkinson MD, Dumontier M, Aalbersberg IJ, Appleton G, Axton M, Baak A, Blomberg N, Boiten J-W, da Silva Santos LB, Bourne PE, et al. The FAIR Guiding Principles for scientific data management and stewardship. Sci Data. 2016;3(1). 10.1038/sdata.2016.18. 10.1038/sdata.2016.18 PMC4792175 26978244

[225] Castro LJ, Palagi PM, Beard N, Attwood TK, Brazas MD. Bioschemas training profiles: A set of specifications for standardizing training information to facilitate the discovery of training programs and resources. PLoS Comput Biol. 2023;19(6):e1011120. 10.1371/journal.pcbi.1011120. 37319143 10.1371/journal.pcbi.1011120 PMC10270332

[226] Gaignard A, Tsueng G, Mičetić I, Castro LJ, Juty N. Improving Bioschemas creation and community adoption through process improvements, tool development, and advancing compliance to FAIR standards. In: Center for Open Science; 2024. 10.37044/osf.io/8km9j.

[227] Tsueng G, Frika S, Gray AJG, Barbero MC, Gaignard A, Mičetić I, Castro LJ, Juty N. Enabling profile updates through the Data Discovery Engine (DDE). In: Center for Open Science; 2023. 10.37044/osf.io/3b9gp.

[228] Gray AJ, Goble CA, Jimenez RC. Bioschemas: From potato salad to protein annotation. In: ISWC (Posters, Demos & Industry Tracks): 2017; 2017.

[229] Tuggle CK, Clarke J, Dekkers JC, Ertl D, Lawrence-Dill CJ, Lyons E, Murdoch BM, Scott NM, Schnable PS. The Agricultural Genome to Phenome Initiative (AG2PI): creating a shared vision across crop and livestock research communities. In*.*, vol. 23: Springer; 2022. p. 1–11. 10.1186/s13059-021-02570-1. 10.1186/s13059-021-02570-1 PMC8722016 34980221

[230] Tuggle CK, Scott N, Clarke J, Murdoch BM, Dekkers JC, Ertl D, Lyons E, Lawrence-Dill CJ, Schnable PS. The AG2pi vision for resources in agricultural genomics and phenomics: How Asas can contribute. J Animal Sci. 2023;101(Supplement_3):50–1. 10.1093/jas/skad281.062.

[231] Yang Z, Fu G, Lee M, Yeo S, Yue GH. Genes for editing to improve economic traits in aquaculture fish species. Aquac Fish. 2024. 10.1016/j.aaf.2024.05.005.

[232] Delomas TA, Hollenbeck CM, Matt JL, Thompson NF. Evaluating cost-effective genotyping strategies for genomic selection in oysters. Aquaculture. 2023;562(738844):738844. 10.1016/j.aquaculture.2022.738844.

[233] Delomas TA, Hollenbeck CM, Matt JL, Thompson NF. Microhaplotypes generate higher breeding value accuracy compared to SNPs for imputation-based breeding strategies. Aquaculture. 2024;586(740779):740779. 10.1016/j.aquaculture.2024.740779.

[234] Palaiokostas C, Houston RD. Genome-wide approaches to understanding and improving complex traits in aquaculture species. CAB Rev Perspect Agric Vet Sci Nutr Nat Resour. 2018;2017(055):1–10. 10.1079/pavsnnr201712055.

[235] Song H, Dong T, Yan X, Wang W, Tian Z, Sun A, Dong Y, Zhu H, Hu H. Genomic selection and its research progress in aquaculture breeding. Rev Aquac. 2023;15(1):274–91. 10.1111/raq.12716.

[236] Boudry P, Allal F, Aslam ML, Bargelloni L, Bean TP, Brard-Fudulea S, Brieuc MSO, Calboli FCF, Gilbey J, Haffray P, et al. Current status and potential of genomic selection to improve selective breeding in the main aquaculture species of International Council for the Exploration of the Sea (ICES) member countries. Aquac Rep. 2021;20(100700):100700. 10.1016/j.aqrep.2021.100700.

[237] Houston RD, Kriaridou C, Robledo D. Animal board invited review: Widespread adoption of genetic technologies is key to sustainable expansion of global aquaculture. Animal. 2022;16(10):100642. 10.1016/j.animal.2022.100642. 36183431 10.1016/j.animal.2022.100642 PMC9553672

[238] Kriaridou C, Tsairidou S, Fraslin C, Gorjanc G, Looseley ME, Johnston IA, Houston RD, Robledo D. Evaluation of low-density SNP panels and imputation for cost-effective genomic selection in four aquaculture species. Front Genet. 2023;14:1194266. 10.3389/fgene.2023.1194266. 37252666 10.3389/fgene.2023.1194266 PMC10213886

[239] Song H, Hu H. Strategies to improve the accuracy and reduce costs of genomic prediction in aquaculture species. Evol Appl. 2022;15(4):578–90. 10.1111/eva.13262. 35505889 10.1111/eva.13262 PMC9046917

[240] Thompson NF, Sutherland BJ, Green T, TA D. A free lunch: microhaplotype discovery in an existing amplicon panel improves parentage assignment for the highly polymorphic Pacific oyster. G3: Genes, Genomes, Genetics. 2025. 10.1093/g3journal/jkae280. 10.1093/g3journal/jkae280 PMC11797050 39700397

[241] Zhou Y, Yang L, Han X, Han J, Hu Y, Li F, Xia H, Peng L, Boschiero C, Rosen BD, et al. Assembly of a pangenome for global cattle reveals missing sequences and novel structural variations, providing new insights into their diversity and evolutionary history. Genome Res. 2022;32(8):1585–601. 10.1101/gr.276550.122. 35977842 10.1101/gr.276550.122 PMC9435747

[242] Tay Fernandez CG, Nestor BJ, Danilevicz MF, Gill M, Petereit J, Bayer PE, Finnegan PM, Batley J, Edwards D. Pangenomes as a resource to accelerate breeding of under-utilised crop species. Int J Mol Sci. 2022;23(5):2671. 10.3390/ijms23052671. 35269811 10.3390/ijms23052671 PMC8910360

[243] Gong Y, Li Y, Liu X, Ma Y, Jiang L. A review of the pangenome: how it affects our understanding of genomic variation, selection and breeding in domestic animals? Journal of Animal Science and Biotechnology. 2023;14(1):73. 10.1186/s40104-023-00860-1. 37143156 10.1186/s40104-023-00860-1 PMC10161434

[244] Golicz AA, Bayer PE, Bhalla PL, Batley J, Edwards D. Pangenomics comes of age: From bacteria to plant and animal applications. Trends Genet. 2020;36(2):132–45. 10.1016/j.tig.2019.11.006. 31882191 10.1016/j.tig.2019.11.006

[245] Kajbaf K, Overturf K, Kumar V. Integrated alternative approaches to select feed-efficient rainbow trout families to enhance the plant protein utilization. Sci Rep. 2024;14(1):3869. 10.1038/s41598-024-54218-2. 38365996 10.1038/s41598-024-54218-2 PMC10873365

[246] Abernathy J, Brezas A, Snekvik KR, Hardy RW, Overturf K. Integrative functional analyses using rainbow trout selected for tolerance to plant diets reveal nutrigenomic signatures for soy utilization without the concurrence of enteritis. PLoS ONE. 2017;12(7):e0180972. 10.1371/journal.pone.0180972. 28723948 10.1371/journal.pone.0180972 PMC5517010

[247] Mengistu SB, Mulder HA, Bastiaansen JW, Benzie JA, Khaw HL, Trinh TQ, Komen H. Fluctuations in growth are heritable and a potential indicator of resilience in Nile tilapia (*Oreochromis niloticus*). Aquaculture. 2022;560:738481. 10.1016/j.aquaculture.2022.738481.

[248] Lallias D, Quillet E, Bégout M-L, Auperin B, Khaw HL, Millot S, Valotaire C, Kernéis T, Labbe L, Prunet P. Genetic variability of environmental sensitivity revealed by phenotypic variation in body weight and (its) correlations to physiological and behavioral traits. PLoS ONE. 2017;12(12):e0189943. 10.1371/journal.pone.0189943. 29253015 10.1371/journal.pone.0189943 PMC5734726

[249] McCarty AJ, Hood S, Huebert K, Cram J, McFarland K, Plough LV. Evaluating a short vs. long-term progeny test and investigating physiology associated with survival in extreme low salinity for the eastern oyster Crassostrea virginica. Aquaculture. 2023;574:739688. 10.1016/j.aquaculture.2023.739688.

[250] McCarty AJ, McFarland K, Small J, Allen S Jr, Plough L. Heritability of acute low salinity survival in the Eastern oyster (*Crassostrea virginica*). Aquaculture. 2020;529:735649. 10.1093/g3journal/jkab368.

[251] Thompson NF, Agnew MV, Calla B, Burge CA. Assessing selection potential for Pacific oyster (*Crassostrea gigas*) to a North American OsHV-1 μvar: Comparing two experimental assay methods. Aquaculture. 2024;590:741076. 10.1016/j.aquaculture.2024.741076.

[252] Vallejo RL, Pietrak MR, Milligan MM, Gao G, Tsuruta S, Fragomeni BO, Long RL, Peterson BC, Palti Y. Genetic architecture and accuracy of predicted genomic breeding values for sea lice resistance in the St John River aquaculture strain of North American Atlantic salmon. Aquaculture. 2024;586(740819): 740819. 10.1016/j.aquaculture.2024.740819.

[253] Wyban J. Selective breeding of *Penaeus vannamei*: Impact on world aquaculture and lessons for future. J Coast Res. 2019;86(sp1):1. 10.2112/si86-001.1.

[254] Houston RD, Haley CS, Hamilton A, Guy DR, Mota-Velasco JC, Gheyas AA, Tinch AE, Taggart JB, Bron JE, Starkey WG, et al. The susceptibility of Atlantic salmon fry to freshwater infectious pancreatic necrosis is largely explained by a major QTL. Heredity (Edinb). 2010;105(3):318–27. 10.1038/hdy.2009.171. 19935825 10.1038/hdy.2009.171

[255] Houston RD, Haley CS, Hamilton A, Guy DR, Tinch AE, Taggart JB, McAndrew BJ, Bishop SC. Major quantitative trait loci affect resistance to infectious pancreatic necrosis in Atlantic salmon (*Salmo salar*). Genetics. 2008;178(2):1109–15. 10.1534/genetics.107.082974. 18245341 10.1534/genetics.107.082974 PMC2248365

[256] Moen T, Torgersen J, Santi N, Davidson WS, Baranski M, Ødegård J, Kjøglum S, Velle B, Kent M, Lubieniecki KP, et al. Epithelial cadherin determines resistance to infectious pancreatic necrosis virus in Atlantic salmon. Genetics. 2015;200(4):1313–26. 10.1534/genetics.115.175406. 26041276 10.1534/genetics.115.175406 PMC4574245

[257] Vallejo RL, Silva RM, Evenhuis JP, Gao G, Liu S, Parsons JE, Martin KE, Wiens GD, Lourenco DA, Leeds TD, et al. Accurate genomic predictions for BCWD resistance in rainbow trout are achieved using low-density SNP panels: Evidence that long-range LD is a major contributing factor. J Anim Breed Genet. 2018;135(4):263–74. 10.1111/jbg.12335. 10.1111/jbg.12335 29869355

[258] Silva RMO, Evenhuis JP, Vallejo RL, Gao G, Martin KE, Leeds TD, Palti Y, Lourenco DAL. Whole-genome mapping of quantitative trait loci and accuracy of genomic predictions for resistance to columnaris disease in two rainbow trout breeding populations. Genet Sel Evol. 2019;51(1):42. 10.1186/s12711-019-0484-4. 31387519 10.1186/s12711-019-0484-4 PMC6683352

[259] Ragone Calvo LM, Calvo GW, Burreson EM. Dual disease resistance in a selectively bred eastern oyster, *Crassostrea virginica*, strain tested in Chesapeake Bay. Aquaculture. 2003;220(1–4):69–87. 10.1016/s0044-8486(02)00399-x.

[260] Moss SM, Moss DR, Arce SM, Lightner DV, Lotz JM. The role of selective breeding and biosecurity in the prevention of disease in penaeid shrimp aquaculture. J Invertebr Pathol. 2012;110(2):247–50. 10.1016/j.jip.2012.01.013. 22434005 10.1016/j.jip.2012.01.013

[261] de Souza Iung LH, Carvalheiro R. Neves HHdR, Mulder HA: Genetics and genomics of uniformity and resilience in livestock and aquaculture species: a review. J Anim Breed Genet. 2020;137(3):263–80. 10.1111/jbg.12454. 31709657 10.1111/jbg.12454

