# Correction: Advancing genetic improvement in the omics era: status and priorities for United States aquaculture

**DOI:** 10.1186/s12864-025-11447-7

**Published:** 2025-03-17

**Authors:** Linnea K. Andersen, Neil F. Thompson, Jason W. Abernathy, Ridwan O. Ahmed, Ali Ali, Rafet Al-Tobasei, Benjamin H. Beck, Bernarda Calla, Thomas A. Delomas, Rex A. Dunham, Christine G. Elsik, S. Adam Fuller, Julio C. García, Mackenzie R. Gavery, Christopher M. Hollenbeck, Kevin M. Johnson, Emily Kunselman, Erin L. Legacki, Sixin Liu, Zhanjiang Liu, Brittany Martin, Joseph L. Matt, Samuel A. May, Caitlin E. Older, Ken Overturf, Yniv Palti, Eric J. Peatman, Brian C. Peterson, Michael P. Phelps, Louis V. Plough, Mark P. Polinski, Dina A. Proestou, Catherine M. Purcell, Sylvie M. A. Quiniou, Guglielmo Raymo, Caird E. Rexroad, Kenneth L. Riley, Steven B. Roberts, Luke A. Roy, Mohamed Salem, Kelly Simpson, Geofrey C. Waldbieser, Hanping Wang, Charles D. Waters, Benjamin J. Reading, The Aquaculture Genomics, Genetics and Breeding Workshop

**Affiliations:** 1 USDA-ARS Aquatic Animal Health Research Unit, Auburn, AL USA; 2 USDA-ARS Pacific Shellfish Research Unit, Newport, OR USA; 3 Department of Animal and Avian Sciences, University of Maryland, College Park, MD USA; 4 Middle Tennessee State University, Murfreesboro, TN USA; 5 USDA-ARS National Cold Water Marine Aquaculture Center, Kingston, RI USA; 6 School of Fisheries, Aquaculture, and Aquatic Sciences, Auburn University, Auburn, AL USA; 7 University of Missouri, Columbia, MO USA; 8 USDA-ARS Harry K Dupree Stuttgart National Aquaculture Research Center, Stuttgart, AR USA; 9 Environmental and Fishery Sciences Division, NOAA Northwest Fisheries Science Center, Seattle, WA USA; 10 Texas A&M AgriLife Research, College Station, TX USA; 11 Texas A&M University - Corpus Christi, Corpus Christi, TX USA; 12 California Sea Grant, Scripps Institution of Oceanography, University of California San Diego, La Jolla, CA USA; 13 Biological Sciences Department, Center for Coastal Marine Sciences, California Polytechnic State University, San Luis Obispo, CA USA; 14 Hubbs-SeaWorld Research Institute, San Diego, CA USA; 15 USDA-ARS National Cold Water Marine Aquaculture Center, Orono, ME USA; 16 USDA-ARS National Center for Cool and Cold Water Aquaculture, Kearneysville, WV USA; 17 Department of Biology, Tennessee Technological University, Cookeville, TN USA; 18 USDA-ARS Warmwater Aquaculture Research Unit, Stoneville, MS USA; 19 USDA-ARS Small Grains and Potato Germplasm Research, Hagerman, ID USA; 20 Harvest Select Catfish Inc, Inverness, MS USA; 21 Washington State University, Pullman, WA USA; 22 Horn Point Laboratory, University of Maryland Center for Environmental Science, Cambridge, MD USA; 23 NOAA Fisheries, Southwest Fisheries Science Center, La Jolla, CA USA; 24 USDA-ARS Office of National Programs, Beltsville, MD USA; 25 Office of Aquaculture, NOAA Fisheries, Silver Spring, MD USA; 26 University of Washington, Seattle, WA USA; 27 School of Fisheries, Aquaculture, and Aquatic Sciences, Auburn University, Alabama Fish Farming Center, Greensboro, AL USA; 28 The Ohio State University, Columbus, OH USA; 29 NOAA Alaska Fisheries Science Center Auke Bay Laboratories, Juneau, AK USA; 30 Department of Applied Ecology, North Carolina State University, Raleigh, NC USA

**Correction: BMC Genomics 26, 155 (2025)**

**https://doi.org/10.1186/s12864-025-11247-z**

Following publication of the original article [1], it was reported that the author Rafet Al-Tobasei was listed with the wrong affiliation and that their affiliation was missing.

The incorrect affiliation was:

Rafet Al-Tobasei^3^

3 Department of Animal and Avian Sciences, University of Maryland, College Park, MD, USA

The correct affiliation is:

Rafet Al-Tobasei^4^

4 Middle Tennessee State University, TN, USA

The correct authorship is available in this Correction and the original article has been updated.
